# Pathways for macrophage uptake of cell-free circular RNAs

**DOI:** 10.1016/j.molcel.2024.04.022

**Published:** 2024-05-17

**Authors:** Laura Amaya, Brian Abe, Jie Liu, Feifei Zhao, Wenyan Lucy Zhang, Robert Chen, Rui Li, Steven Wang, Roarke A. Kamber, Miao-Chih Tsai, Michael C. Bassik, Ravindra Majeti, Howard Y. Chang

**Affiliations:** 1 Center for Personal Dynamic Regulomes, Stanford University, Stanford, CA 94305, USA.; 2 Institute for Stem Cell Biology and Regenerative Medicine, Stanford University School of Medicine, Stanford, CA, CA 94305, USA.; 3 Division of Immunology and Rheumatology, Department of Medicine, Stanford University, Stanford, CA 94305, USA.; 4 Department of Medicine, Division of Hematology, Stanford University School of Medicine, Stanford, CA, 94305, USA.; 5 Department of Genetics, Stanford University School of Medicine, Stanford, CA 94305, USA.; 6 RNA Medicine Program, Stanford University, Stanford, CA 94305, USA.; 7 Howard Hughes Medical Institute, Stanford University, Stanford, CA 94305, USA.

**Keywords:** circRNA, MSR1, phagocytosis, macropinocytosis

## Abstract

Circular RNAs (circRNAs) are stable RNAs present in cell-free RNA, which may comprise cellular debris and pathogen genomes. Here we investigate the phenomenon and mechanism of cellular uptake and intracellular fate of exogenous circRNAs. Human myeloid cells and B cells selectively internalize extracellular circRNAs. Macrophage uptake of circRNA is rapid, energy-dependent, and saturable. CircRNA uptake can lead to translation of encoded sequences and antigen presentation. The route of internalization influences immune activation after circRNA uptake, with distinct gene expression programs depending on the route of RNA delivery. Genome-scale CRISPR screens and chemical inhibitor studies nominate macrophage scavenger receptor MSR1, toll-like receptors, and mTOR signaling as key regulators of receptor-mediated phagocytosis of circRNAs, a dominant pathway to internalize circRNAs in parallel to macropinocytosis. These results suggest that cell-free circRNA serves as an “eat me” signal and danger-associated molecular pattern, indicating orderly pathways of recognition and disposal.

## eTOC Blurb

Amaya et. al. explores how cells take up extracellular circular RNAs (CircRNAs) and their impact on immune signaling. Macrophages readily internalize circRNAs, and this study identifies the specific receptors and signaling pathways governing circRNA internalization, highlighting their role as signaling molecules for immune recognition and disposal.

## Graphical Abstract

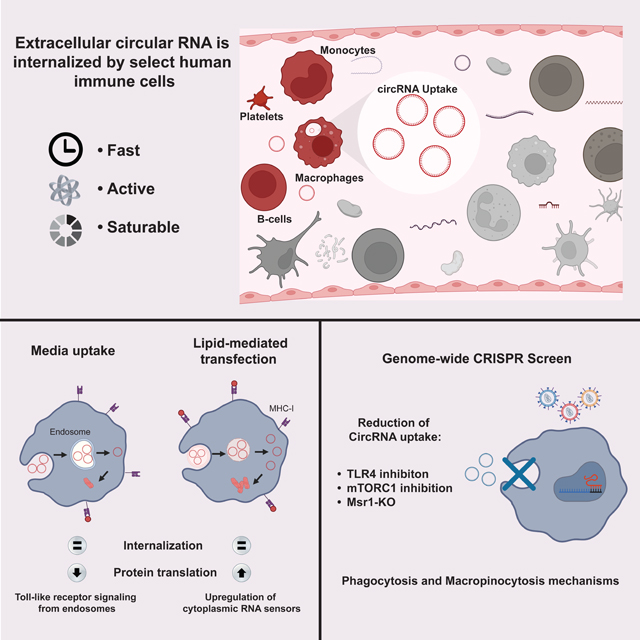

## INTRODUCTION

Circular RNAs (circRNAs) are covalently closed, single-stranded loops formed in human cells through a non-canonical splicing process called back-splicing. CircRNAs are distinct from linear messenger RNAs because circRNAs lack 5’ and 3’ ends, making circRNAs resistant to degradation by exoonucleases^1^. Although circRNAs resemble messenger RNA (mRNA) in their extended length, spanning from hundreds to tens of thousands nucleotides, circRNAs can exhibit half-lives over several days, while their linear mRNAs typically have shorter half-lives, usually less than 20 hours^1^. The covalent joining of the two ends may also promote circRNAs to adopt complex secondary and tertiary structures, which differentiates it from double-stranded molecules such as plasmid DNA (pDNA), double-stranded (dsRNA), or small interfering (siRNA) duplexes that lack similar shapes. Several circRNAs are known to serve essential biological functions, by acting as microRNA sponges^2^, inhibiting proteins^3^, regulating the activity of proteins^4^, and even serving as templates for translation^5^. Due to their spatio-temporal and tissue-specific expression patterns and prolonged stability, circRNAs hold promise as potential biomarkers for human diseases^6^. Cell-free circRNA has been identified in various bodily fluids, including human peripheral whole blood, plasma, saliva, and urine^7,8,9^. Additionally, circRNAs have been found to be present and stable in exosomes^10^.

Circular RNAs are prevalent in human and pathogen genomes. The human genome encodes tens of thousands of endogenous circRNAs, many of them being isoforms of mRNA genes. Moreover, many pathogens have circular RNA genomes, including hepatitis D virus and viroids that can extensively infect plants and bacteria^11^. Recent studies have revealed a system of innate immunity distinguishing self vs. foreign circRNAs^12–14^. Unlike some endogenous circRNAs that act as suppressors of innate immunity^12^, exogenous circRNAs can induce potent immune signaling^13^. Innate immunity of circRNA has been leveraged to create circRNA based vaccines with in vivo efficacy against infectious disease and cancer^14,15^. The nucleic acid sensor, RIG-I, can detect exogenous circRNAs based on differences of an RNA modification and trigger a robust antiviral immune response^16^. However, RIG-I is located in the cytoplasm. This observation implies either the existence of an undiscovered cell surface sensor for circular RNA or the existence of a mechanism that facilitates the entry of circular RNA into the interior of antigen presenting cells. It is at present a mystery how immune cells would survey the repertoire of circRNAs from dead or dying cells.

It has been known for over 30 years that select cells can take up and express naked RNAs in vivo^17^. One of the main challenges when using nucleic acids as therapeutics is to achieve efficient intracellular delivery. Although nucleic acids generally cannot readily cross cell membranes because of their size and charge^18^, specific entry routes have been identified for the uptake of plasmid DNA^17^, DNA oligonucleotides^19^, siRNA^20^, dsRNA^21^, and mRNA^22^, involving different endocytic pathways and diffusion-controlled mechanisms in a cell type-specific manner. Moreover, numerous animal species can uptake in vitro transcribed dsRNA from the environment to initiate RNA interference^23^. Professional phagocytes such as dendritic cells (DCs) and macrophages have been shown to internalize mRNA, and in preclinical vaccine development; the immune effects on dendritic cells were mediated by the recognition of mRNA by TLR4 and TLR7^24^. Classic studies suggested that DCs primarily internalize mRNAs through micropinocytosis, where extracellular material in fluid phase are engulfed in bulk. In contrast, macrophages can take up plasmid DNA through scavenger receptors and receptor-mediated phagocytosis^25^. Building on these findings, at least five naked mRNA-based vaccines have been reported^26–28^. In contrast to the extensive studies of mRNA uptake, whether and how cell-free circRNA -a far more stably species--is internalized are poorly understood.

RNA uptake is distinguished from therapeutic RNA delivery via transfection. Endocytic uptake results in RNA being internalized into endosomes, and an additional step is required for RNA to escape into cytosol for translation. Therapeutic RNA delivery is often aimed at delivering RNA directly into the cytosol for efficient translation. Nonetheless, the two concepts can converge. The majority of lipid nanoparticle-siRNA complexes are taken up by endocytosis, and only a small fraction of RNAs escape from endosomes^29^. Similarly, GalNac-modified siRNAs are taken up by receptor-mediated endocytosis and slowly released from endosomes for sustained therapeutic effect.

In the course of studying circRNA vaccination, we found that systemic administration of naked exogenous circRNA can elicit potent antigen-specific immune responses in mice^15^. Subcutaneous administration of purified cell-free circRNA, which were circularized through the self-splicing Td intron from T4 bacteriophage, led to uptake in macrophages and dendritic cells and trafficked to draining lymph nodes and spleen^26^. These intriguing observations were at the organismal level. However, the precise mechanism responsible for this adjuvant effect remains enigmatic. Here, we aim to investigate the cellular mechanisms and immune responses associated with the path of uptake of cell-free circRNA. By elucidating these aspects, we can gain valuable insights into the interplay between circRNA properties, immune responses, cellular uptake mechanisms, which may inform circRNA biology and development of RNA therapeutic strategies. In this study we reveal previously unrecognized pathways through which cell-free circular RNA can be taken up by macrophages. This observation not only illuminates the enigmatic mechanisms driving the adjuvant properties of circular RNA but also holds substantial implications for the advancement of the RNA therapeutics field.

## RESULTS

### Cell-type specific uptake of extracellular circular RNAs

**Figure 1.**
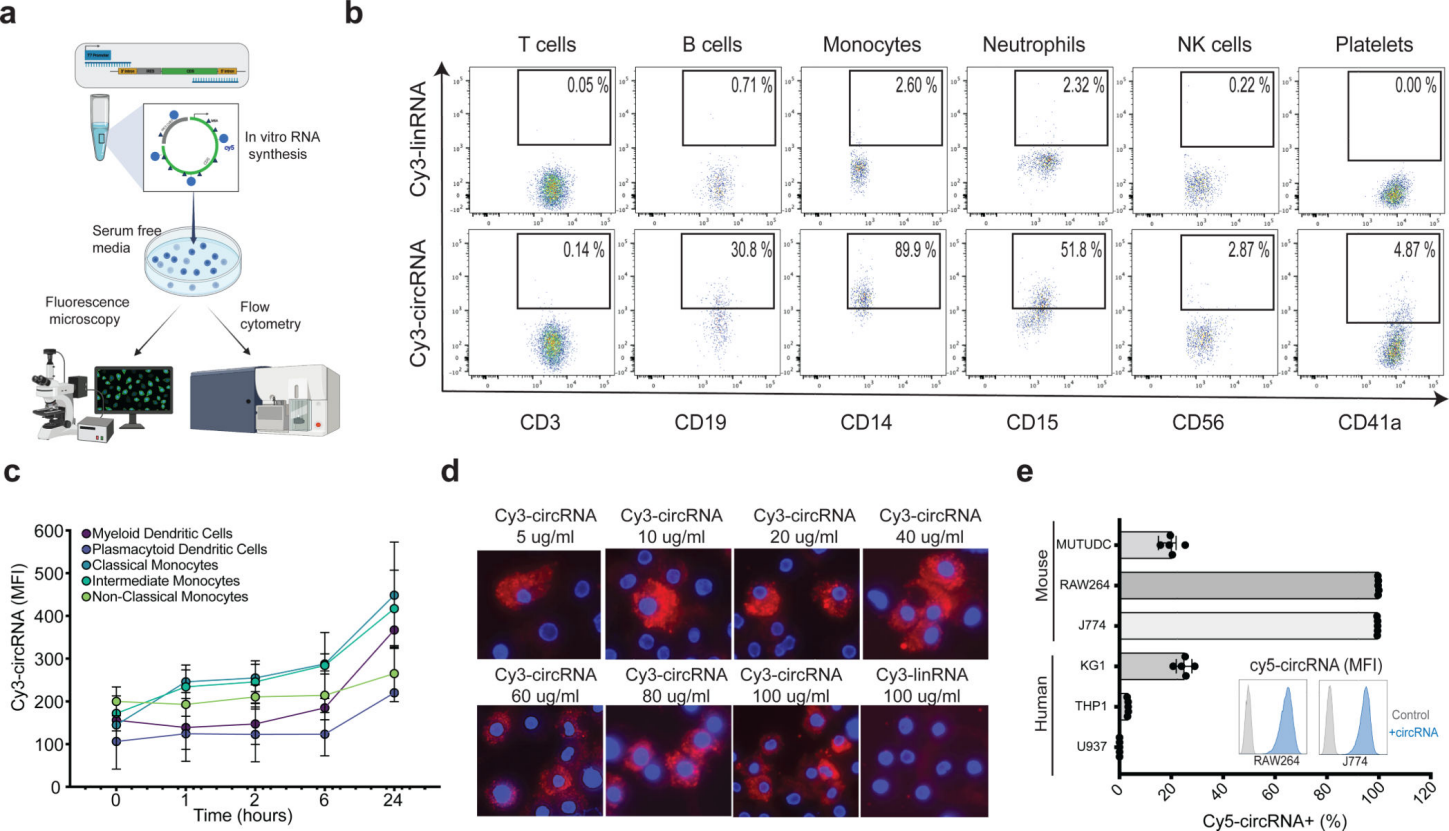
Extracellular circular RNA uptake is cell specific. **a,** Schematic representation of experimental model for circRNA uptake measurements. circRNA are in vitro transcribed with fluorescent UTP and added into serum free media to already confluent cells in culture, which are then analyzed by fluorescence microscopy or flow cytometry. **b,** CircRNA and linRNA uptake measured by flow cytometry in distinct hematopoietic cell subsets from human peripheral blood after 2 hours incubation. Percentage of Cy3+ out of each cell type is displayed (representative sample of two independent experiments) **c,** Fluorescent intensity of human myeloid immune subsets after distinct incubation time points with fluorescently labeled circRNA (n = 3, bars represent SEM). **d,** Fluorescent microscopy of labeled circRNA (increasing concentrations) and linRNA (last image) in human macrophages after 2 hours incubation. Cy3 (red) and DAPI (blue). **e,** Percentage of fluorescently labeled cells after circRNA uptake in mouse (top) and human (bottom) cell lines (n = 5, bars represent SEM). Histogram representation of fluorescent intensity of negative control compared to 100 ng of circRNA (representative sample in J774 and RAW264 cell lines). NK = natural killer cells. See also Figure S2.

CircRNA is internalized by macrophages and dendritic cells in vivo when delivered subcutaneously in mice^15^. To study the mechanisms of this phenomenon, we first investigated the potential of human blood cells to uptake circRNA after adding naked circRNA to primary human blood cells in vitro (passive pulsing). We produced and purified in vitro transcribed circRNA as previously described^16^, (Supplementary Table 1) with the incorporation of fluorescently labeled UTPs (Figure 1a). To verify efficient circularization, we performed circRNA quantification via RT-qPCR with divergent primers that span the splice junction and only amplifies product when circRNA is present (Supplementary Figure 1a). To assess the purity of the circular RNA products and the absence of small dsRNA contaminants, we have conducted Tape Station analysis (Supplementary Figure 1b) and demonstrated the purity of the in vitro transcribed circular RNA samples and absence of linear contaminants after RNase R digestion. Moreover, we measured the presence of dsRNA, as a major contaminant of in vitro-transcribed (IVT) mRNA, which can induce severe anti-viral immune responses. Our results confirm that none of our circular RNA payloads contain levels of dsRNA contaminants (<0.01ng/ug of RNA prep) that could lead to immune cell activation (Supplementary Figure 1c). We minimized the possibility of dye-specific uptake by swapping different fluorescent dyes such as Cy5, Cy3 and FITC throughout our experiments, and similar results were obtained. Primary human peripheral blood mononuclear cells (PBMCs) were isolated from healthy donors and incubated for 2 hours with fluorescently labeled circRNA in serum-free medium, which prevents circRNA binding to albumin^30^, and cellular uptake of circRNA was quantified by flow cytometry. We observed uptake of Cy3-labeled circRNA (Cy3-circRNA) in monocytes, neutrophils, B cells, and platelets based on the expression of the cell surface markers CD14, CD15, CD19 and CD41a, respectively, on Cy3+ cells (Figure 1b). Notably, the uptake of circRNA by monocytes was substantially elevated compared to the remainder of the cell types examined, with ~90% of monocytes becoming Cy3+ after 2 hours. We repeated this experiment using a linear RNA format with the exact same sequence and length, lacking a cap and poly(A) tail but maintaining comparable stability to circRNA in serum-free media for up to 24h (Supplementary Figure 1d, e). Surprisingly, the cellular uptake of Cy3-labeled linear RNA (Cy3-linRNA) in any of the PBMC subsets was 20- to 40-fold lower. While cell-free linear RNA has the same stability as circRNA under the assay conditions, this experiment does not rule out the possibility of greater circRNA stability over linear RNA upon cellular internalization. Closer examination of specific human myeloid subsets revealed that classical monocytes (defined here as CD14+CD16-) exhibited higher circRNA uptake throughout a 24-hour time course (Figure 1c, Supplementary Figure 2a, b). These results indicate that several human immune cell types take up extracellular circRNAs, consistent with our prior observations made during naked circRNA delivery in mice^15^.

### Macrophages efficiently take up circRNA in a dose dependent manner

We next imaged circRNA uptake via fluorescence microscopy, which can distinguish RNA internalization compared to cell surface binding and provides an orthogonal validation to flow cytometry. Considering that classical monocytes can differentiate into macrophages, which are specialized phagocytic cells, we next examined uptake and cellular localization of circRNA by human primary macrophages. Different concentrations of Cy3-circRNA were incubated with macrophages for 2 hours. The macrophages exhibited a dose-dependent uptake of Cy3-circRNA, reaching saturation at 40 ug/ml (Figure 1d). This observation aligns with the findings from the FACS-based uptake assay conducted under identical conditions (Supplementary Figure 2c). Cy3-circRNA was detected in the cytoplasm of human macrophages, and no uptake was detected with an equimolar amount of Cy3-linRNA, which served as a negative control (Figure 1d). We observed that individual macrophages appear to exhibit different capabilities of circRNA uptake. At low doses of naked circRNA, single macrophages take up circRNA that fill vesicles in its cytoplasm preferentially over its neighbors. At higher doses, more macrophages in the population start to take up circRNAs.

To establish a baseline model to study the molecular dynamics of circRNA uptake, we proceeded to measure circRNA uptake in a panel of human and murine myeloid cell lines. CircRNA uptake levels were cell line-specific, and the strongest circRNA uptake was observed in differentiated macrophage lines mouse RAW264 and J774, human KG-1, and mouse dendritic cells line MutuDC (Figure 1e). Low levels of circRNA uptake were observed in monocyte-like human cell lines, THP1 and U937. CircRNA uptake was concentration dependent as observed in both human classical monocytes (Supplementary Figure 2d) and macrophages cell lines J774 and RAW264 (Supplementary Figure 2e). Because many RNA therapeutics localize to liver hepatocytes when delivered *in vivo*^31^, we also evaluated circRNA uptake in tissue-specific cell lines, and observed strong uptake of Cy5-labeled circRNA (Cy5-circRNA) in the human hepatoma cell line HepG2, lung epithelial cell line Calu-3, and lung fibroblasts IMR-90 (Supplementary Figure 2f).

### CircRNA uptake by macrophages is fast, saturable, and energy dependent.

**Figure 2.**
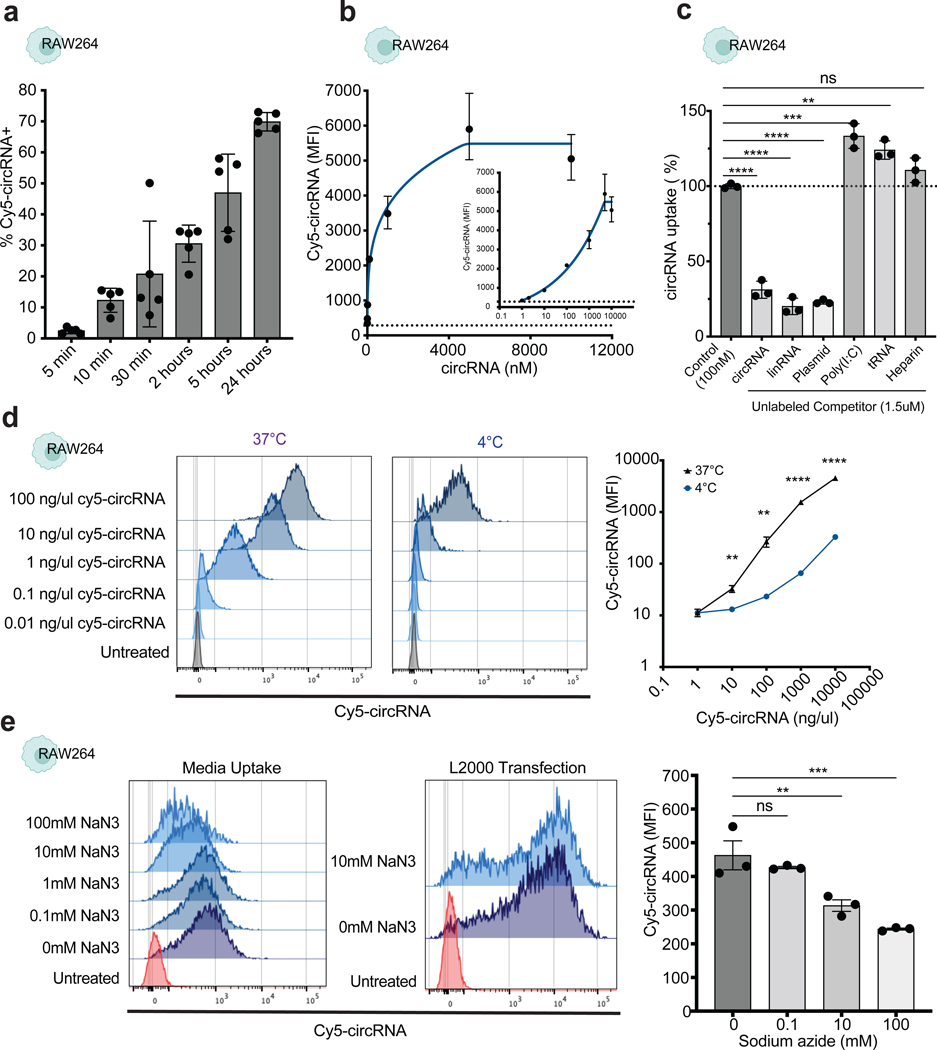
Circular RNA uptake is a fast and active process. **a,** Time course analysis of circRNA uptake measured by flow cytometry in RAW264 cells (n = 5, bars represent SEM). **b,** Saturation curve of circRNA uptake in RAW264 cells after 24 hours incubation. Logarithmic and linear scale displayed for accurate visualization (n = 3, bars represent SEM). **c,** Competitor assay showing fold change of circRNA uptake percentage after normalization to Cy5-circRNA only control (100 nM) in RAW264 cells (n = 3, bars represent SEM). The dose of each competitor (1.5 uM) used in these experiments is 15 times higher to ensure complete saturation of cell receptors. **d,** Fluorescent intensity of RAW264 cells after incubation or lipofectamine transfection with fluorescently labeled circRNA at increasing concentrations comparing temperature effect (representative sample on the left and summary quantification on the right) (n = 4, bars represent SEM). Statistical significance was calculated using one sample t and Wilcoxon test **P < 0.001, ****P < 0.0001. **e,** Increasing concentration of sodium azide effect on circRNA uptake in RAW264 cells (representative sample on the left and summary quantification on the right) (n = 3, bars represent SEM). One-way ANOVA followed by Tukey’s test was applied in c and e, **P < 0.01, ***P < 0.001, ****P < 0.0001. ns = not significant. See also Figure S3.

To understand the potential mechanisms of circRNA uptake we measured the kinetics of circRNA internalization using the macrophage cell line RAW264, as this cell line showed the strongest signal after passive pulsing of circRNA. Uptake of Cy5-circRNA was observed immediately after 5 min, and continued to accumulate for 24 hours, indicating a fast and continuous process (Figure 2a). The percentage of fluorescently positive cells increased linearly over time (*R*^2^ = 0.7805, *p* value = 0.0196, y = 0.03877x + 18.17), which may suggest the involvement of a fluid-phase endocytosis mechanism. Additional hallmarks of receptor-mediated endocytosis are (i) saturation at high ligand concentrations and (ii) specific competition for uptake by an excess of the same or related ligands. When uptake of circRNA was analyzed at increasing concentrations of Cy5-circRNA, we observed a saturable process that plateaus at 1 uM circRNA concentration (Figure 2b). To determine the specificity of circRNA uptake, we performed a competition assay. Unlabeled circRNA, linear RNA or plasmid DNA efficiently competed for Cy5-circRNA uptake (Figure 2c). In contrast, Poly(I:C), which is structurally similar to double-stranded RNA, and tRNA did not compete with circRNA uptake and instead had a positive effect in circRNA uptake. In addition, heparin, which is comparable in mass and charge to some oligonucleotides, failed to interfere with circRNA uptake. These results suggest that macrophages take up circRNAs via a saturable and ligand-selective process, likely involving one or more receptors.

To assess whether circRNA is internalized via an active or diffusion controlled mechanism, we examined the temperature dependence of circRNA uptake. A cellular uptake process is termed active if it requires energy. This is typically determined by inhibiting energy production with cold temperature or metabolic blockade. CircRNA uptake was severely inhibited at 4°C compared to 37°C incubation across multiple ligand concentrations (Figure 2d), suggesting that circRNA uptake in RAW264 cells is energy dependent. CircRNA uptake was also substantially blunted at 4°C in HepG2, IMR-90, and Calu3 cells (Supplementary Figure 3a). When RAW264 cells were treated with ATP inhibitor, sodium azide, circRNA uptake was substantially reduced (Figure 2e) and a similar effect was observed in primary macrophages (Supplementary Figure 3b). In contrast, we observed no effect in circRNA internalization when Cy5-circRNA was delivered by lipid-mediated transfection in RAW264 cells (Figure 2e). Similar results were confirmed with other metabolic inhibitors such as oligomycin that inhibits ATP synthase, and 2-deoxyglucose that interferes with d-glucose metabolism (Supplementary Figure 3c). Conversely, we tested if we could promote circRNA uptake by inducing macrophage activation and consequently enhancing phagocytosis. Both PMA and LPS stimulation of RAW264 macrophage cells resulted in a significant increase of Cy5-circRNA uptake (Supplementary Figure 3d). These results suggest that macrophage uptake of extracellular circRNA is an active and regulated process. Just as mRNA uptake has been studied and characterized in dendritic cells, we replicated our findings regarding circRNA uptake in mouse dendritic cells, also revealing a rapid and energy-dependent mechanism in dendritic cells (Supplementary Figure 3e, f).

To quantify circRNA uptake in the absence of fluorescent dyes, we performed RT-qPCR in RAW264 cells after 24-hour incubation with unlabeled circRNA using primers that target specifically the back splice regions of circRNA. CircRNA levels after uptake were comparable to lipofectamine transfection (Supplementary Figure 3g). Uptake of linear RNA was also detectable by RT-qPCR (Supplementary Figure 3g), a more sensitive assay than measuring fluorescence. CircRNA uptake was greater than linRNA uptake in RAW264 and J774 cells but comparable in HepG2 cells (Supplementary Figure 3h). To track internalization of circRNA in live cells, we used circRNA that was covalently bound to the intracellular pH indicator pHrodo-Red, a fluorogenic probe that is weakly fluorescent at neutral pH and increasingly fluorescent in acidic conditions. We observed a close correlation of pHrodo-Red signal and Cy5 fluorescence (Supplementary Figure 3i). These results corroborate intracellular localization of circRNA and suggest that some fraction of circRNA is localized in acidic compartments such as endosomes or lysosomes.

### CircRNA uptake by dendritic cells results in translation and presentation to T cells.

Another indication of circRNA internalization is the potential for protein translation. CircRNA needs to traverse the cell membrane and enter the cytosol in order to interact with the translation machinery. To determine if circRNA can be translated after being taken up, we designed two circRNA molecules, one encoding a model antigen, chick Ovalbumin (hereafter named circOVA), and the other encoding the reporter protein Nanoluciferase (hereafter named circNanoLuc). Since circRNAs lack a 5′ end, they rely on cap-independent translation mediated by internal ribosome entry sites (IRES). To maximize circRNA translation, we used previously optimized elements for circRNA-encoded protein production^32^. These elements include optimized RNA chemical modifications, 5’ and 3’ untranslated regions, HRV-B3 IRES, and synthetic aptamers shown to increase circRNA translation over mRNA after a single transfection.

**Figure 3.**
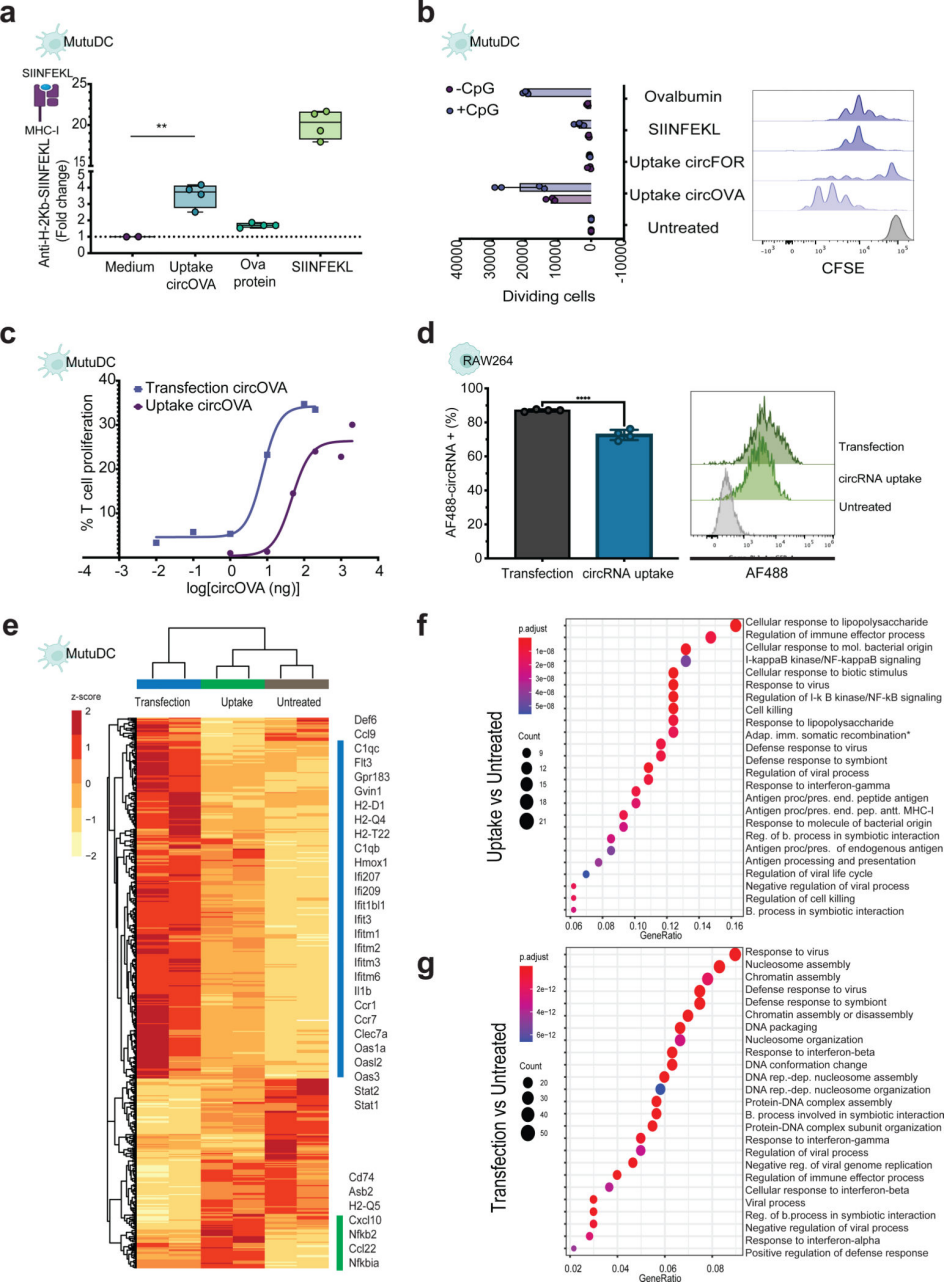
Protein expression after circular RNA uptake compared to cell transfection. **a,** Fluorescent intensity of SIINFEKL bound to H-2Kb of MHC class I antibody compared to control after 24 incubation with circOVA, OVA protein, or SIINFEKL control in MutuDC cells (n = 4, bars represent Min and Max). Statistical significance was calculated using one-way ANOVA followed by Tukey’s test, **P < 0.01. **b,** Proliferation assay comparing antigen specific T cell proliferation level of OT-I cells co-cultured with MutuDC cells incubated with OVA protein, SIINFEKL, and circOVA or non-translatable circRNA control (representative sample on the right and summary quantification on the left) (n = 4, bars represent SEM). **c,** circOVA titration with and without lipid-mediated transfection to determine the minimum amount required to induce antigen-specific T cell proliferation. **d**, Percentage of fluorescently labeled cells after circRNA uptake in RAW264 cells (n = 4, bars represent SEM). Histogram representation of fluorescent intensity of negative control compared to 100 ng of circRNA uptake or transfection (representative sample). **e,** Heatmap of normalized expression data showing differentially regulated genes following circRNA uptake or circRNA transfection compared to untreated cells. Gene expression is shown in normalized log2 counts per million. Differentially expressed genes were selected based on a > 2-fold change and padj value < 0.05. Functional analysis of the top differentially expressed genes and their linkages with biological concepts (GO terms) after **f,** circRNA uptake and **g,** circRNA transfection. See also Figure S4.

To determine if circRNA can be translated and processed for antigen presentation by dendritic cells, we tested the antigen presentation capacity of MutuDCs after incubation with circOVA. Using specific MHC tetramers, we measured ovalbumin-derived peptide SIINFEKL bound to H-2Kb of MHC class I on MutuDC cells after 24 hours incubation with circOVA, SIINFEKL peptide, or OVA protein. Incubation of MutuDC cells with circOVA in the media resulted in 5-fold increase of OVA peptide antigen presentation compared to control (Figure 3a). Furthermore, we measured the capability of antigen-primed dendritic cells to induce T-cell specific proliferation *in vitro*. CircOVA uptake in MutuDC cells resulted in the strongest induction of antigen-specific T-cell proliferation after 3-day co-culture with OT-1-transgenic CD8 T cells, even above OVA protein or SINFEKL peptide incubation (Figure 3b). This effect was enhanced after addition of CpG oligodeoxynucleotides, short synthetic single-stranded DNA, known to induce dendritic cell maturation and to maximize antigen capture and presentation^33^. These findings provide evidence that circRNA can undergo cellular uptake and translation in the cytosol, followed by processing of the encoded protein, which can subsequently be presented to the immune system. To further investigate this phenomenon, we conducted a T-cell proliferation assay, using varying amounts of circOVA delivered with or without a transfection reagent into MutuDC cells. We observed a remarkable ~100-fold reduction in the amount of input material needed to induce antigen-specific T-cell proliferation when circRNA was transfected, compared to the naked uptake method (Figure 3c).

After passive pulsing of circNanoLuc in RAW264 cells, circRNA translation was comparable to mRNA-NanoLuc (Supplementary Figure 4a). This observation was replicated in HepG2 cells after incubation with increasing concentrations of the corresponding mRNA or circRNA encoding nanoluciferase (Supplementary Figure 4b). Transfection and uptake of J774 cells with both mRNA and circRNA expressing Nanoluciferase resulted in robust protein translation. This translation efficiency was notably higher compared to the untranslatable circRNA control (circSTOP), which harbors a stop codon at the very beginning of its coding sequence (Supplementary Figure 4c). When comparing the protein translation levels of circNanoLuc after uptake vs transfection in RAW264 cells, we observed that the transfection of circNanoLuc leads to 10-fold higher luminescence signal compared to passive pulsing of circRNA (Supplementary Figure 4d), however when comparing the levels of circRNA internalization levels, transfection is just 15% higher than circRNA uptake (Figure 3d). These results indicate that only a small portion of circRNA is readily available for translation after uptake.

### Path of circular RNA entry activates distinct transcriptional programs

To further investigate the mechanisms behind the differences observed in naked circRNA uptake vs. lipid-mediated transfection, we compared the transcriptome profile of cells after 24 hrs of these two conditions by RNA-seq. Principal component analysis (PCA) revealed uptake vs. transfection of circRNA led to dominant and distinct transcriptome profiles based on the path of circRNA entry (Supplementary Figure 4e). CircRNA uptake was characterized by upregulation of *Nfkbia* and *Nfkb2*, two canonical genes indicative of NF-kB pathway activation, and *Cd74*, a critical chaperone in antigen processing, which directs transport to the endosomal/lysosomal system (Figure 3e). On the other hand, circRNA transfection resulted in significant upregulation of *Hmox1*, a membrane-bound enzyme with cytoprotective effects, in addition to upregulation of several interferon induced transmembrane proteins (Figure 3e). Differential expression analysis between treatments revealed specific upregulation of cell membrane components involved in substrate recognition and antigen processing and presentation (*Cd74, Asb2, Mhc-I* splice variants) in the ‘circRNA uptake’ group, whereas ‘circRNA transfection’ resulted in significant upregulation of cytoplasmic RNA sensors (*Dhx58, Oas1g, Oas2*) (Supplementary Figure 4f). Gene Ontology of differentially upregulated genes after circRNA uptake showed significant association with “Cellular response to LPS” (Figure 3f), suggestive of Toll-like receptor signaling from endosomes. Conversely, the transcriptional profile of transfected cells was significantly enriched for “Response to virus” and dsRNA signaling from cytosol (Figure 3g). We have recently shown the recognition of circRNA in vivo by the innate cell compartment, and showed circRNA uptake leads to DC maturation and upregulation of CD80, CD86, MHC class I and II^15^. However, the effects of innate immune sensing on different formats of circRNA delivery are incompletely understood.

Our GO enrichment analysis suggests that naked circRNA is localized/detected in the cell membrane or endosomal compartments, whereas circRNA delivered with a transfection reagent might be localized in the cytosol and readily available for translation. These results indicate that depending on the internalization method, circRNA is localized to distinct cellular compartments, and thus is recognized and processed differently. These results might also explain the low translation efficiency after naked circRNA uptake. Only when the circRNA is translated into proteins and be degraded by proteosomes in the cytoplasm, then the antigenic peptide will be transported to the Endoplasmic reticulum (ER) and loaded onto MHC class I molecules^34^. Nonetheless, both treatments lead to activation of viral responses and immune effector processes. Toll-like receptor will recognize circRNA localized to endosomes/lysosomes and RIG-I-like receptors will sense circRNA in the cytosol. Both signaling pathways lead to induction of proinflammatory cytokines and type I interferons. When analyzing the transcriptome regulation in RAW264 cells after circRNA uptake, we observe similar activation of Type I interferon signaling, and activation of cytoplasmic RNA sensors. (Supplementary Figure 4g, h). Furthermore, we didn’t observe any gene expression changes related to cellular metabolism, cell cycle control and apoptosis, due to transfection mediated toxicity^35^. We repeated this experiment in J774 cells and included our circSTOP as untranslatable control. The results from this experiment (Supplementary Figure 4i, j) demonstrate that despite the lack of protein production, the immune response pathways remained consistent, suggesting that the recognition of circRNA by the immune system is not solely dependent on protein expression.

### CRISPR screen identifies regulators of circRNA uptake

**Figure 4.**
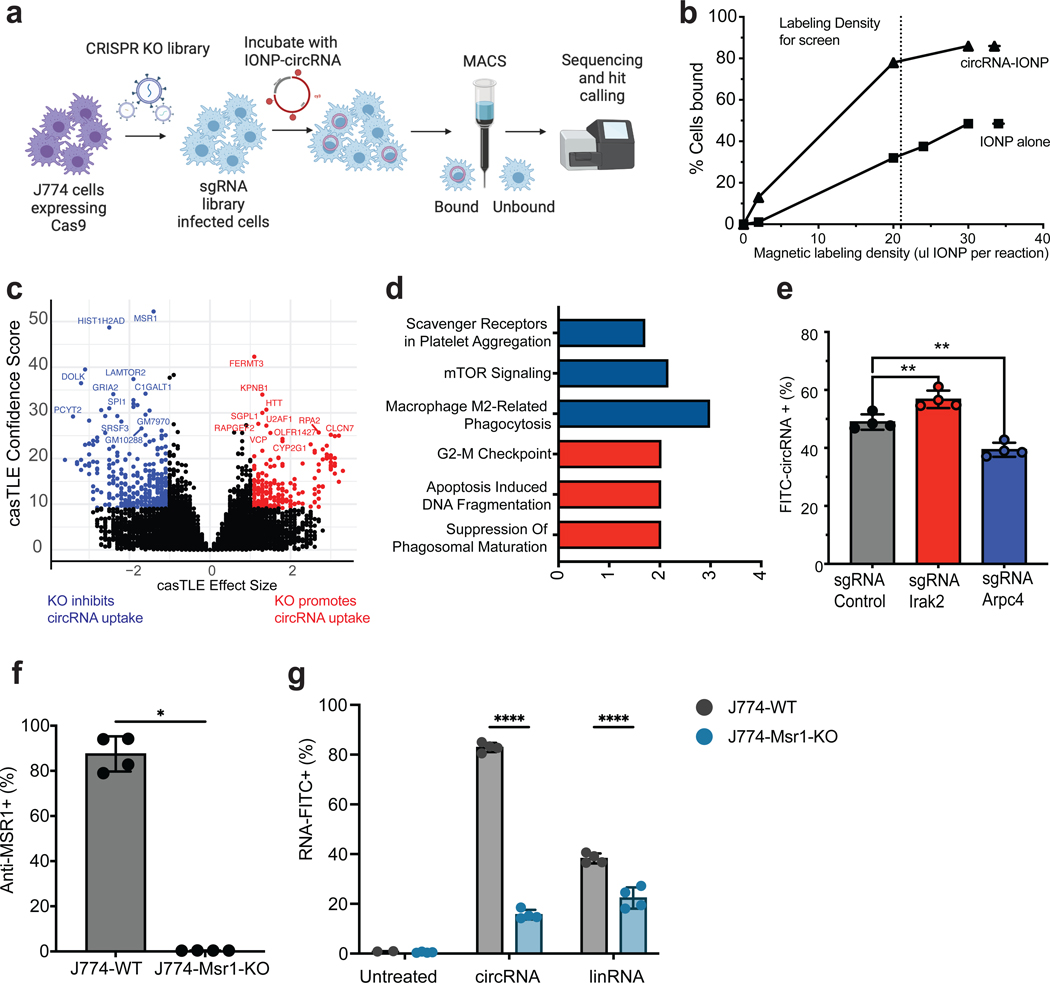
CRISPR screen identifies regulators of circRNA uptake. **a**, Schematic representation of circRNA uptake screening using magnetic separation. **b**, Percentage of J774 cells bound to magnet with IONP alone or when IONP is covalently bound to circRNA. Dotted line indicates the concentration of IONP used for labeling reaction. **c**, Volcano plot of all genes indicating effect and confidence scores for genome-wide circRNA uptake screen. Effect and confidence scores determined by casTLE. **d,** Select Reactome categories enriched in the 46 genes that pass the 10% FDR cutoff as determined by casTLE. Categories include: Reactome 2022 and Elsevier Pathway Collection. **e**, CircRNA uptake of single KOs in J774-Cas9 cells after 6 hours incubation with 1 ug/ul of FITC-circRNA. Positive regulator in blue, negative regulator in red. (n = 3, bars represent SD). Statistical significance was calculated using a two-tailed unpaired t-test. **P < 0.01. **f,** Quantification of MSR1 protein levels assessed by antibody staining of surface receptors, after Msr1 KO in J774-Cas9 cells (n = 4, bars represent SD). Statistical significance was calculated using a two-tailed unpaired t-test. *P < 0.05. **g,** Percentage of circular and linear RNA uptake in Msr1 KO cells (n = 4, bars represent SD). Statistical significance was calculated using a two-way ANOVA, ****P < 0.0001. See also Figure S5.

Research has shown that inhibiting macropinocytosis results in the cessation of RNA engulfment by DCs following intranodal injection^22^. However, in numerous studies conducted thus far, accurately differentiating between macropinocytosis and receptor-mediated endocytosis as the mechanisms for antigen uptake in DCs has proven challenging^36^. The molecular mechanisms of RNA uptake by immune cells remain enigmatic. In contrast to using chemical inhibitors, which can often be nonspecific and lead to cell toxicity, we deployed a pooled CRISPR screening approach to systematically investigate the essential requirements for circRNA uptake in macrophages^37,38^. By stably expressing Cas9 in J774 cells, we generated a pool of gene knockouts by introducing a lentiviral library of single-guide RNAs (sgRNAs) targeting every protein-coding in the genome. J774-Cas9 cells were separated based on their capacity to take up circular RNA (Figure 4a). We found that circRNA acts as a macrophage uptake signal when coupled to foreign objects. Covalent conjugation of purified circRNA to 30 nM-diameter iron oxide nanoparticles (IONP, Methods) led to avid uptake of the nanoparticles, allowing macrophages that ingested the circRNA-IONP to be enriched by magnetic cell sorting (MACS) (Figure 4b). In contrast, a lower percentage of binding was observed when using IONP-mRNA, as depicted in Supplementary Figure 5a. We envision this experimental design mimics cell-free circRNAs present in large complexes of RNAs and RNA binding proteins^37^. After 24-hour incubation with magnetic-circRNA, cells were passed through a uniform magnetic field that captured magnetized cells which ingested the magneticcircRNA. A direct comparison of sgRNA abundance in each sorted fraction (magnet-bound vs unbound) was performed after genomic DNA isolation and sequencing. Gene-level effects were then derived using casTLE as previously described^38^. We performed two counter screens using either (1) unconjugated IONP particles or (2) IONP particles conjugated to linear mRNA, and removed hits that were identified in either counter screen. The counter screen with IONP alone did not yield significant hits after multiple comparison correction (Supplementary Table 2). IONP-mRNA screen yielded only two gene hits at false discovery rate (FDR<10%, *Plod3, P4hb*) that overlapped genes regulating circRNA uptake (Supplementary Figure 5b, Supplementary Table 3); these two genes were not characterized further as they are not circRNA specific.

We identified 46 genes that specifically promoted or inhibited circRNA uptake (FDR<10%, Figure 4c, Supplementary Table 4). Pathway analysis for the genes discovered as positive hits in both screens showed strong enrichment of Reactome pathway terms related to scavenger receptors, phagocytosis, and mTORC signaling. Among the most prominent negative regulators of circRNA uptake were genes associated with apoptosis and DNA replication, which might enhance circRNA accumulation when perturbed (Figure 4d). To validate the effects of individual genes, we generated stable J774 cell lines expressing Cas9 and specific sgRNAs targeting the identified hits. Subsequently, we quantified circRNA uptake using flow cytometry. Notably, J774 cells expressing sgRNAs against *Arpc4,* a regulator of actin polymerization essential for formation of the phagocytic cup^39^, displayed reduced circRNA uptake, whereas sgRNAs targeting *Irak2,* a key kinase downstream of TLR receptors signaling to NFkB^40^, resulted in increased circRNA uptake (Figure 4e). Both of these results are consistent with their observed phenotypes in the pooled screens.

### A receptor-mediated phagocytosis pathway for circRNA uptake

**Figure 5.**
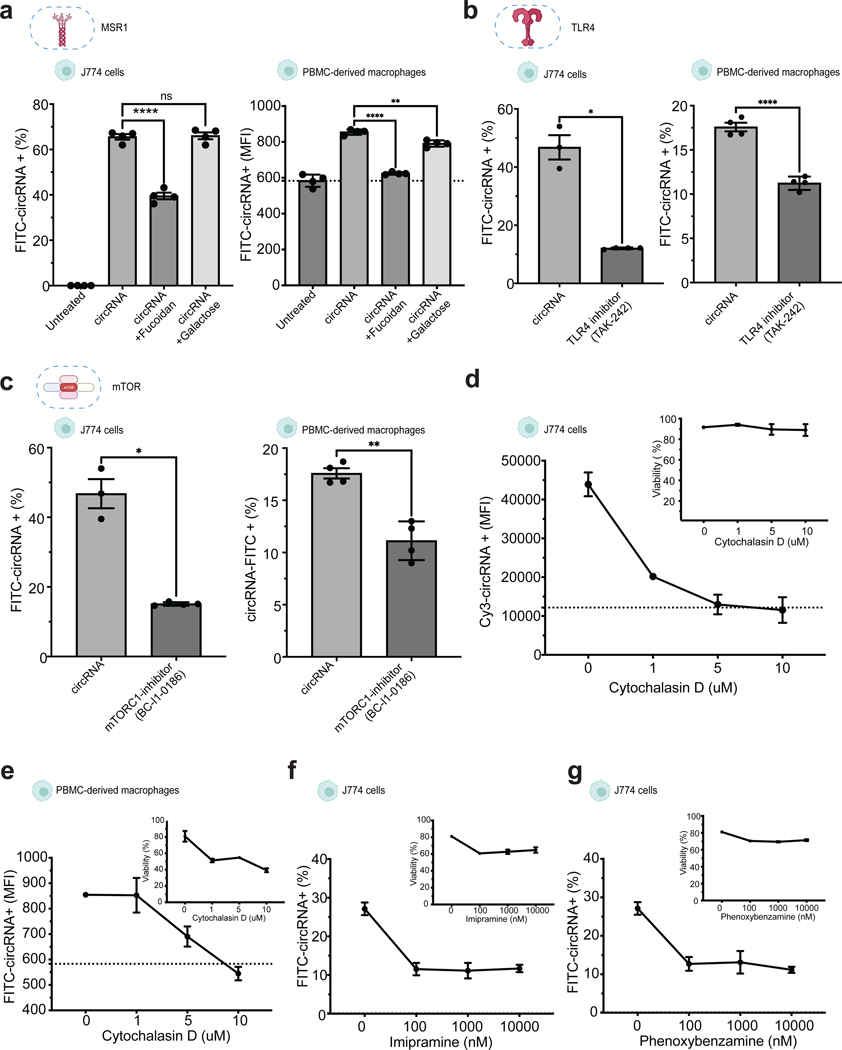
Validation of circRNA uptake regulators with chemical inhibitors. **a**, Competition assay of circRNA and scavenger receptor ligands in J774 cells (left) and human primary macrophages (right), (n = 3, bars represent SD). Statistical significance was calculated using one-way ANOVA **b**, Chemical inhibition of TLR4 effect in circRNA uptake in J774 cells (left) and primary human macrophages (right). **c**, Chemical inhibition of TLR4 in circRNA uptake in J774 cells (left) and primary human macrophages (right). (n = 3, bars represent SD). **d,** Concentration-dependent effects of cytochalasin D on circRNA uptake in J774 cells and viability after treatment (top right). (n = 3, bars represent SEM). **d**, Chemical inhibition of mTORC1 in circRNA uptake in J774 cells (left) and primary human macrophages (right). (n = 3, bars represent SD). **e,** Concentration-dependent effects of cytochalasin D on circRNA uptake in primary human macrophages and viability after treatment (top right), (n=3, bars represent SEM). **f,** Concentration-dependent effects of imipramine and **g,** phenoxybenzamine on circRNA uptake in J774 and viability after treatment (n = 3, bars represent SEM). Statistical significance was calculated using a two-tailed unpaired t-test. Differences between groups were considered significant for P values < 0.05. See also Figure S6.

Genes enriched in the unbound fraction led to decreased circRNA uptake when disrupted, and therefore are normally necessary for efficient circRNA internalization (‘positive regulators’). The top hit was *Msr1*, encoding the class A macrophage scavenger receptor (SR-A) that is a major phagocytic receptor known to bind diverse ligands including dsRNA and is critical to activate TLR signaling in endosomes^41^ (Figure 4c). *Spi1*, a transcriptional regulator of *Msr1* expression^42^, was also among the top hits (Figure 4c). To validate the involvement of *Msr1* in circRNA uptake, we generated a J774 cell line with *Msr1* knockout (*Msr1*-KO) and assessed the impact on circRNA uptake. Our results clearly indicated a significant reduction in circRNA uptake in the *Msr1*-KO cells compared to wild-type cells, thus confirming the role of MSR1 in facilitating circRNA internalization (Figure 4f, g). To examine the specificity of MSR1 for circRNA vs. linear RNA, we evaluated uptake of labelled cell-free linear RNA with 5-fold greater specific activity (dye molecules/ug RNA), to increase sensitivity of linRNA uptake detection. Msr1 proficient J774 macrophages exhibited 2-fold lower linear RNA uptake than circRNA uptake, and Msr1-KO had a substantially lower magnitude of decrement on linear RNA uptake (Figure 4g). These results suggest that Msr1 is a preferential receptor for circRNA uptake. As additional validation, we performed competition experiments with fluorescently labeled circRNA. The MSR1 ligand fucoidan binds to and inhibit MSR1-mediated uptake in a competitive fashion while the structurally related sugar galactose does not bind MSR1. Passive pulsing of FITC-circRNA in the presence of fucoidan but not galactose significantly inhibited circRNA internalization in both J774 cells and primary human macrophages (Figure 5a).

Scavenger receptors and TLRs are known to act in coordination in generating innate immune responses^43^. TLR family members individually signal to MSR1 to direct macrophage phagocytic activity toward specific pathogens or PAMPs^44^. Correspondingly, when we inhibited TLR4, we observed a significant downregulation of circRNA uptake in both J774 cells and primary macrophages (Figure 5b), indicating an involvement of toll like receptor in circRNA uptake. We also identified members of the Ragulator complex, such as Lamtor2, as required mediators of circRNA uptake (Figure 4c). Lamtor2 plays a vital role in macrophages’ ability to combat Salmonella infection by regulating replication within the phagosome^45^. Moreover, Lamtor2 is critically involved in mTOR signaling transduction, activating mTOR and impacting endosomal biogenesis and receptor trafficking^46^. When we specifically blocked mTOR complex 1 by chemical inhibitors, we observed a significant decrease in the uptake of fluorescent-circRNA in both J774 cells and primary human macrophages (Figure 5c). Inhibitors of TLR4 or mTORC1 did not affect linear RNA uptake of J774 cells (Supplementary Figure 6a, b), indicating that both TLR and mTORC1 contribute specifically to circRNA uptake.

Conversely, sgRNAs enriched in the bound fraction led to increased circRNA uptake when disrupted, and therefore normally inhibit circRNA internalization (‘negative regulators’). One of the top genes was *Fermt3* (Figure 4c) which is a leukocyte adhesion molecule of the Kindlin family that regulates macrophage phagocytosis and motility^47^. The previously mentioned *Irak2* may be another negative regulator due to the known ability of pro-inflammatory signaling to inhibit *Msr1* transcription and activity in macrophages^41^. Thus the candidate negative regulators emerging from the CRISPR screen reinforce the idea that circRNA uptake is regulated and require cytoskeletal remodeling.

### Macropinocytosis also contributes to circRNA uptake

To investigate the involvement of actin polymerization in circRNA internalization, we treated J774 cells with cytochalasin D, a drug that disrupts actin filaments. We observed a concentration-dependent inhibition of circRNA uptake in both J774 cells and primary macrophages (Figure 5d, 5e). However, cytochalasin D is known to affect both phagocytosis and macropinocytosis mechanisms^48^. To further distinguish between these mechanisms, we employed imipramine and phenoxybenzamine, potent inhibitors of macropinocytosis that do not exhibit cytotoxic effects or hinder other endocytic pathways^49^. We found that circRNA uptake was reduced by half with all concentrations of each inhibitor tested, although complete inhibition was not observed (Figure 5f, g). Additionally, we assessed the effects of knockouts of *Arpc4* and *Irak2*, a positive and negative regulator of circRNA uptake respectively, on the uptake of other substrates, such as dextran (a marker for macropinocytosis) and transferrin (a marker of clathrin-mediated endocytosis) (Supplementary Figure 6c). Interestingly, only the disruption of *Arpc4* had a negative impact on dextran uptake, which is known to be internalized in an actin-dependent manner. Thus, a subset of the regulators of circRNA uptake may be general regulators of macropinocytosis. Furthermore, we evaluated the impact of all tested chemical inhibitors on different form of nucleic acids, including mRNA and plasmid DNA (Supplementary Figure 6d, e). Our results indicate distinct responses to circRNA compared to other nucleic acids in the presence of chemical inhibitors. Particularly, we observed slight increase in fluorescence following DNA uptake. However, upon treatment with cytochalasin D or specific micropinocytosis inhibitors (imipramine and phenoxybenzamine), we observed no reduction in fluorescence, indicating incomplete internalization of DNA. This suggests that while the DNA may interact with the cell membrane, it is not fully internalized.

**Figure 6.**
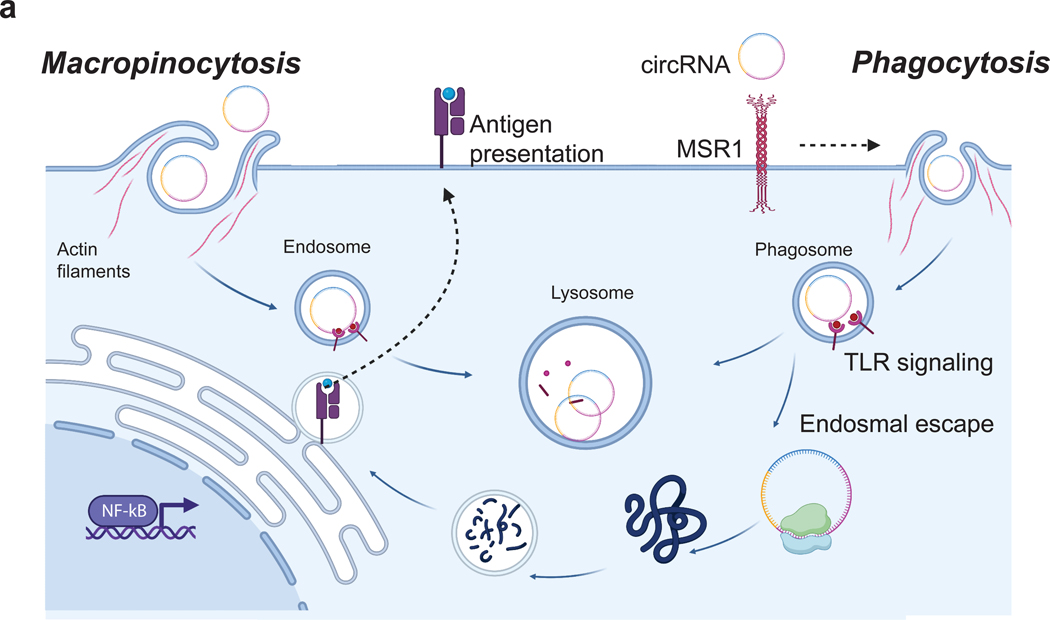
Mechanisms of circRNA uptake. **a,** Cartoon representation of circRNA uptake mechanisms in macrophages. MSR1 triggers the internalization of circRNA. This internalization process initiates both phagocytic and macropinocytic activities, facilitating the transport of circRNA to degradative endosomal/lysosomal compartments. Within these compartments, circRNA engages with TLR receptors, triggering an immune response. Notably, a fraction of the internalized circRNA manages to escape into the cytosol, leading to protein synthesis and facilitating subsequent antigen presentation.

Based on the combination of our screen results and chemical inhibition studies, we suggest that circRNA uptake is facilitated by endocytosis mechanisms involving actin polymerization, possibly through a combination of receptor-mediated phagocytosis via the scavenger receptor MSR1 and constitutive macropinocytosis (fluid-phase endocytosis) in macrophages (Figure 6).

## DISCUSSION

### Antigen presenting cells take up cell-free circRNA

CircRNA represents an attractive platform as biomarkers and vectors for gene expression due to its superior durability and stability. However, the extent to which extracellular circRNAs may engage with innate immune cells are poorly understood. The intrinsic stability of circRNAs raises the intriguing concept that an active clearance system may exist to take up, process, and respond to extracellular circRNAs. In this study, we explore the uptake of cell-free circRNA by human and mouse cells, and we demonstrate that circRNA uptake is a cell-specific and energy-dependent process. Notably, primary monocytes exhibited selective uptake of circRNA, and this uptake was significantly enhanced in differentiated mouse macrophages and dendritic cells. The selectivity of circRNA uptake by specialized immune subsets, such as antigen-presenting cells (APCs), enabled us to explore the molecular and functional consequences of circRNA recognition. Through transcriptome analysis, we showed that circRNA encoding antigen can induce immune activation, in a manner strongly dictated by the path of circRNA internalization.

### Potential mechanisms for circRNA internalization

We identified the involvement of the scavenger receptor MSR1 in the internalization of extracellular circRNA through a combination of genomic screening and validation experiments using chemical inhibition. We envision that MSR1 binding leads to receptor-mediated phagocytosis of the circRNA, which is then transported to degradative endosomal/lysosomal compartments, where it activates TLR receptors. Notably, a portion of the internalized circRNA manages to escape into the cytosol, leading to protein synthesis and facilitating subsequent antigen presentation (Figure 3b). A parallel pathway of RNA uptake involves macropinocytosis, previously implicated in dendritic cells. Macropinocytosis occurs in response to cellular stimulation, leading to the formation of ruffles on the cell surface that engulf extracellular material in the fluid phase into vesicles. This process implies that any circRNA, regardless of its association with an activating receptor, has the potential to be internalized by macropinocytosis. Chemical inhibitor studies suggest a role for macropinocytosis in circRNA uptake as well. There are many overlapping properties between macropinocytosis and phagocytosis, e.g. actin dependence, and the relationship between these two pathways is open for future studies. Classic studies of mRNA uptake reported predominantly dendritic cell involvement but limited activity for macrophages^22,24^. These results may reflect the intrinsic instability of mRNAs or the differential activity of dendritic cells vs. macrophages. These results provide mechanistic insights into the internalization mechanisms of exogenous circRNA. Moreover, the implications of MSR1 upregulation in various diseases^41^ open up exciting possibilities for utilizing the ligand binding and endocytic capabilities of this receptor to specifically deliver disease-modifying drugs to macrophages. This potential application holds promise for targeted and effective therapeutic interventions.

The successful delivery of therapeutic mRNA relies on its efficient escape from endosomes, a pivotal step in the process. Several mechanisms have been proposed to elucidate this crucial event. Notably, early endocytic/recycling compartments have been identified as primary sites for mRNA escape. Additionally, various mechanisms including pore formation, pH-buffering, and fusion with the lipid bilayer of endosomes have been suggested to facilitate mRNA release^50^. The ‘proton sponge’ hypothesis suggests that cationic polyplexes induce osmotic swelling, aiding in endosomal escape^51^. Furthermore, recent attention has been drawn to the role of mRNA localization to the endoplasmic reticulum, which contributes to evading stress granule sequestration^52^. Collectively, these studies underscore the intricate nature of endosomal escape, which involves a repertoire of potential mechanisms warranting further investigation after circRNA uptake.

### Implications of circRNA uptake for physiology and disease

Cell-free circRNA has been documented as powerful biomarkers of human diseases^53^, and our work reveals an intercellular perspective for circRNA processing and turnover. Our studies also raise the intriguing possibility that endogenous circRNAs may underlie local and long-range communications between organs and the immune system. The release of endogenous circRNAs from cell death or secretion may also activate the immune system, in an analogous fashion to the delivery of exogenous circRNAs. We and others have previously studied circular RNA translation after cytosolic delivery via RNA transporters designed to facilitate the passage of RNA across cell membranes. The fact that naked circRNA possesses the ability to penetrate cells, impact intercellular signaling, and translated for antigen presentation underscores the unique characteristics and potential applications of this class of RNAs. These areas may be fruitful directions for future investigations.

### Limitations of the study

In this work we synthesized and purified circRNA ex vivo for uptake by immune cells. RNAs are likely to be associated with RNA binding proteins in the form of ribonucleic protein complexes^16^, which may alter the circRNA uptake process or kinetics. We used CRISPR knockout screen to identify genes and pathways that regulate circRNA uptake. Genes that act redundantly will be missed in such single knockout screens. Dual targeted screens may identify additional regulators and refine their epistatic relationships in the future. Finally, while we surmise that internalized circRNA can activate TLR signaling in endosomes, we this study did not address the cytosolic escape of internalized circRNAs.

## STAR METHODS

### RESOURCE AVAILABILITY

#### Lead contact

Further information and requests for resources and reagents should be directed to and will be fulfilled by the lead contact, Howard Y. Chang (howchang@stanford.edu).

#### Materials availability

All unique reagents and materials reported in this study will be made available upon reasonable request without restrictions.

#### Data and code availability

- RNA-seq data are available at Gene Expression Omnibus (GEO) under accession number GSE264160. These data are publicly available as of the date of publication.
- This paper does not report original code.
- additional information required to reanalyze the data reported in this paper is available from the lead contact upon request.

#### Experimental Model and Study Participant Details

##### Cell lines

The RAW264.7 (TIB-71) and J774A.1 (donated by Bassik Laboratory at Stanford University) cell lines were purchased from ATCC and cultured in DMEM medium supplemented with 10% FBS and 1% penicillin/streptomycin (Thermo Fisher). Cells were passaged after reaching 90% confluence, detached with cell scraper, and subcultured at 1:8 ratio every two days. U937 (donated by Bassik Laboratory at Stanford University) and THP-1 (TIB-202) cells were acquired from ATCC. Cells were maintained in suspension culture with RPMI 1640 medium supplemented with 2mM glutamine, 10% FBS, and 1% penicillin-streptomycin. Cells were subcultured when cell concentration reached 8×105 cells/mL.

MutuDC cells were purchased from Applied Biological Materials Inc. (abm T0528). Cells were maintained in IMDM-Glutamax (Gibco 31980) medium supplemented with 10% FBS, 1% penicillin-streptomycin, 10 mM Hepes (Gibco 15630), and 50 μM β-mercaptoethanol (GIBCO 31350). KG-1 (CCL-246) cells were acquired from ATCC. Cells were maintained in DMEM medium supplemented with 20% FBS and 1% penicillin-streptomycin. Calu-3 (HTB-55), IMR-90 (CCL-186), and Hep G2 (HB-8065) cells were acquired from ATCC and maintained in EMEM medium supplemented with 10% FBS, 1% penicillin-streptomycin. For routine subculture, 0.25% Trypsin-EDTA (Thermo Fisher) were used for cell dissociation. All cell lines were kept in culture at 37°C in a humidified incubator with 5% CO2, and regularly tested for mycoplasma contamination (Lonza LT07–318).

### METHOD DETAILS

#### CircRNA design and in vitro transcription

CircRNA templates were synthesized by cloning DNA fragments into a custom entry vector which contains self-splicing introns, 5’ PABP spacer, HBA1 3’ UTR, and HRV-B3 IRES. CircRNA were synthesized using HiScribe T7 High Yield RNA Synthesis Kit (NEB E2040S) IVT templates were PCR amplified (Q5 Hot Start High-Fidelity 2x Master Mix) and column purified (Zymo DNA Clean & Concentrator-100) prior to RNA synthesis as previously described^13,15,32^. Briefly, 1 ug of circRNA PCR-template was used per 20 μL IVT reaction. Reactions were incubated overnight at 37°C. IVT templates were subsequently degraded with 2 μL of DnaseI (NWB M0303S) for 20 minutes at 37°C. The remaining RNA was column purified and digested with 1U of RnaseR per microgram of RNA for 60 minutes at 37°C. Samples were then column purified, quantified using a Nanodrop One spectrophotometer, and verified for complete digestion using an Agilent TapeStation. When compared to mRNA or linRNA, the same sequence was used as IVT template with the addition of 100bp poly(A) tail incorporated after the 3’ UTR. mRNA was synthesized using the same IVT kit and the addition of 4nM CleanCap AG (TriLink N-7113). CircRNA, linRNA or mRNA were fluorescently labeled by incorporating 5% of Fluorescein-12-UTP (Sigma-Aldrich 11427857910) in the corresponding IVT reaction, or by post-transcriptional modification using Label IT Nucleic Acid Labeling Kit (Mirus Bio Cy3, Cy5, Fluorescein, or AF488) at 1:25 (Dye:RNA). When comparing circRNA uptake to mRNA or linRNA we used 1:5 (Dye:RNA) to increase the fluorescent signal. Three different circRNA were produced, circOVA which encodes OVA protein, circNanoLuc which encodes nanoluciferase protein, and circFOR that has a shift-frame mutation that interferes with protein translation. Both circOVA and circNanoLuc were enhanced for translation by adding 5% m6A modifications and 5% of 2’OMeC.

#### CircRNA uptake by human PBMCs

Human peripheral blood mononuclear cells (PBMCs) were isolated from buffy coat obtained from female healthy donors at Stanford Blood Center on a Ficoll-Paque gradient. 10 ng/ul of circRNA either unlabeled or Cy3 labeled was added to 1X10^5^ cells in RPMI-1640 medium and incubated at 37°C for 2 hours. Subsequently, the cells were washed with FACS buffer and stained with anti-CD14 (AF647, BioLegend 325611), anti-CD3 (AF647, BioLegend 300422), anti-CD19 (AF647, BioLegend 302222), anti-CD56 (AF647, BioLegend 318313), anti-CD15 (AF647, BioLegend 323012), or anti-CD41a (AF647, BioLegend 303725). After 30 minutes incubation on ice, the cells were then washed with FACS buffer and stained with DAPI. Uptake of circRNA in each cell subtype was analyzed by flow cytometry. Unlabeled and Cy3-linear RNA were used as negative controls.

#### CircRNA uptake by human monocytes

Peripheral blood mononuclear cells were prepared from whole blood from healthy volunteers using Lymphoprep (StemCell Technologies 07801) following the manufacturer’s protocol. Briefly, whole blood was diluted 1:1 in complete media (RPMI + 2% FBS-HI (Heat Inactivated at 65°C for 30 min) and carefully layered onto an equal volume of Lymphoprep (15ml), centrifuged at 800g for 30min at room temperature with brakes off, and buffy coat was carefully transferred to a fresh vial. After washing once with complete media, red blood cells were lysed at 4°C for 10min, the cells were washed in complete media twice, and cells were frozen using a 1:1 dilution in freezing media (90% FBS-HI, 10% DMSO).

Frozen PBMC were thawed, washed in media, and viability assessed using a Countess automated cell counter (ThermoFisher). 1×10^6^ cells in 50 ul were aliquoted into each well of a 96-well plate, an equal volume of cy3-labeled circRNA dilutions. Cells were placed at 37°C 5% CO2 for the indicated time points. At each time point, the cells were transferred to a fresh 96 well V-bottom plate and washed with FACS buffer. Cells were incubated with the following at room temperature protected from light: ZombieRed live/dead stain (Biolegend 423110) for 10 min, Human TruStain FcX (Biolegend 422302) for 5 min, and the following antibody mixture for 30min: anti-CD3e (FITC, Biolegend 300406), anti-CD11c (BV711, Biolegend 301629), anti-HLADR (BV650, Biolegend 307650), anti-CD16 (BV510, Biolegend 360733), anti-CD123 (BV421, Biolegend 306017), anti-CD19 (AF700, Biolegend 302225), anti-CD14 (AF647, Biolegend 325611). Cells were washed twice in FACS buffer and fixed in 4% PFA for 30min. The fixed cells were washed with FACS buffer and stored at 4ºC until analyzed by flow cytometry.

#### CircRNA uptake by human macrophages

Human primary macrophages were differentiated as described previously^59^. Briefly, human PBMCs were enriched for monocytes using EasySep Human Monocyte Enrichment Kit without CD16 Depletion (STEMCELL Technologies 19059). The resulting monocytes were resuspended in IMDM Glutamax in the presence of 10% Human Serum and 1X Penicillin-Streptomycin at a density of 1×10^6^ cells/mL and cultured in a tissue culture dish at 37ºC for 6–7 days to obtain differentiated macrophages.

Aliquot 2×10^5^ differentiated macrophage cells in 100 ul medium in a 24-well plate and add circRNA to the final concentrations as indicated. After incubation for 2hr, the cells were harvested and cytospinned on the glass slides. The cells were stained by DAPI and the localization of the circRNA was analyzed under fluorescence microscopy.

#### CircRNA uptake and transfection

Cell lines were seeded at 1×10^5^ cells per well in a 96-well plate in complete media. After 24 hours cells were washed twice with serum-free media and circRNA was added at 1ug/ul or at the indicated concentrations. Cells were placed at 37°C and 5% CO2 for 18h or the indicated time points. Media containing circRNA was then removed, cells were transferred to a v-bottom plate and washed twice with PBS, stained with Live/dead NIR fixable dye and analyzed by flow cytometry. To reduce background surface binding, we perform a mild trypsin treatment before every flow cytometry analysis, to ensure the removal of membrane bound dyes. CircRNA or mRNA transfection was performed using TransIT-mRNA transfection kit (Mirus MIR 2250), with 3 μL of TransIT-mRNA reagent (Mirus Bio) per microgram of RNA.

#### Inhibitors and promoters of circRNA uptake

RAW264 cells were seeded as previously described and incubated with 100 nM cy3-circRNA in serum free media in combination with either: 1.5uM of unlabeled circRNA, linRNA, plasmid DNA, Poly(I:C) (Sigma-Aldrich P9582), tRNA (Sigma-Aldrich TRNABAK-RO) or 5ug/ml of heparin (Sigma-Aldrich H3149). After 24-hour incubation, cells were analyzed by flow cytometry as previously described. RAW264 cells were seeded as previously described and treated with increasing concentrations of sodium azide (Sigma-Aldrich S2002). After 30 min pre-incubation, with sodium azide, cells were washed twice with PBS and 1ug of Cy5-circRNA was added into serum-free media or transfected into cells. 24 hours after treatment, cells were analyzed by flow cytometry as previously described. RAW264 cells were seeded as previously described and treated for 30 min with 20mM of 2-Deoxy-D-glucose (Tocris 4515) or 2 mg/ml of oligomycin (Sigma-Aldrich O4876). Cells were then washed twice with PBS and 1ug of Cy5-circRNA in serum-free media was added onto cells. RAW264 cells were seeded as previously described and 1ug/ul of Cy5-circRNA in serum-free media was added onto cells in combination with 20 ng/ml of LPS (Sigma-Aldrich L4516), or 5 ng/ml of PMA (Sigma-Aldrich P1585). 24 hours after treatment, cells were analyzed by flow cytometry as previously described.

For validation of mechanisms of circRNA uptake, J774 cells and primary human macrophages were incubated with 1 ug/ml of FITC-circRNA in combination with 4 mg/ml of Fucoidan or Galactose. Similarly, before incubation with circRNA, J774 cells and primary human macrophages were pre-treated for 15 min with different chemical inhibitors: TLR4 inhibitor (10 nM), mTORC1 inihbotor (50 nM), Cytochalasin D (1–10uM), Imipramine (100 nM to 10 uM), and Phenoxybenzamine (100 nM to 10 uM). Similarly, we compared circRNA uptake with uptake of Dextran (100 ug/ml) and Tranferrin (5 ug/ml) after individual gene KOs.

#### In vitro NanoLuciferase assay

Cells were harvested at 24 hours post-uptake or transfection in 100 μL of passive lysis buffer (Promega) and lysed by rocking and pipetting for roughly 15 minutes at room temperature. Lysate was centrifuged at 4,000 rcf for 10 minutes to clear debris, and 5 μL of clarified lysate was transferred into a 384-well white-bottom assay plate (Perkin Elmer). To each well, 10 μL of ONE-Glo EX from the Promega Nano-Glo Dual-Luciferase Reporter Assay System was added, after which the plate was vortexed for 1 minute, incubated at room temperature for an additional 2 minutes, and read on a TECAN Infinite Pro microplate reader.

#### T cell proliferation assay

OT-I CD8 T cells were purified from TCR-transgenic mice OT-I by negative selection using immunomagnetic beads (Miltenyi Biotech). For direct MHC-I antigen presentation assays, MutuDC lines were seeded at 10,000 cells per well in round-bottom 96-well plates. For MHC-I-restricted antigen presentation assays, MutuDC were incubated for 2 h with 1nM SIINFEKL (OVA257–264, Sigma-Aldrich S7951), 1mg/ml of Ovalbumin protein (InvivoGen vac-pova), 1ug of circFOR, or 1ug of circOVA, in the presence or absence of 1uM CpG (ODN 1585, InvivoGen). Cells were washed three times in medium and incubated with 50,000 purified OT-I CD8 T cells (CFSE-labeled). T cell proliferation was measured after 60 h of culture by flow cytometry analysis excluding doublets and dead cells. OT-I CD8 T cells were gated as CD8+ Vα2+ cells. Live dividing T cells were detected as low for cell proliferation dyes (CFSE low). MutuDC were similarly transfected with circOVA with or without transfection reagent at the indicated concentrations.

#### RNA-seq and transcriptome data analysis

MutuDC cells were incubated with naked circRNA as previously described or transfected with CART-circRNA complex. After 24 hours of incubation, total RNA was extracted from the cells using the RNeasy Mini Kit (Qiagen) according to the manufacturer’s protocol. The integrity of the total RNA was analyzed using an Agilent 2100 Bio-analyzer and RIN numbers were above 9. RNA libraries were prepared with the Illumina Ribo-Zero Plus rRNA Depletion Kit. The adaptor ligated libraries were sequenced using an Illumina NextSeq 500. Transcript abundances were calculated by pseudocounts using Salmon (version 1.4.0). Normalization and differential gene expression analyses were performed by the DESeq2 package (v1.32.0) in R (version 4.1.1). Enrichment analysis for GO terms were performed by the ClusterProfiler package (v4.0.5) and graphs were produced by the ggplot2 package (v3.3.5).

#### CRISPR library preparation

The 10-sgRNA-per-gene CRISPR/Cas9 deletion library was transduced into Cas9-expressing J774 cells as previously described^60^. Briefly ~300 million J774 cells stably expressing Cas9 were infected with the ten-guide-per-gene genome-wide sgRNA library at a multiplicity of infection <1. Infected cells underwent puromycin selection (1 μg/ml) for 5 d, after which puromycin was removed and cells were resuspended in normal growth medium without puromycin. After selection, sgRNA infection was measured as >90% of cells as indicated by measuring mCherry-positive cells with flow cytometry. Sufficient sgRNA library representation was confirmed by deep sequencing after selection.

#### Individual sgRNA-expressing J774

For generating individual sgRNA (Arpc4 and Irak2) phenotypes we lentivirally infected J774 cells stably expressing Cas9 with constructs expressing a given sgRNA along with puromycin resistance. At 3 days after infection, cells were selected with 5 ug/ml puromycin for an additional 3 days, and allowed to recover for 3 days before use in assays.

Cells were harvested, and total genomic DNA was extracted using QuickExtract DNA Extraction Solution (VWR, QE09050). PCR amplification was carried out with primers designed approximately 250–350 bp upstream and 450–600 bp downstream of the predicted cut site, followed by Sanger sequencing to confirm knock-out.

A similar approach was employed to generate J774-Msr1-KO cells, utilizing five distinct sgRNAs targeting exon 1. Following selection, single-cell clones were expanded to ensure the purity of cell populations. Sequencing analysis confirmed deletions leading to a premature stop codon, and the complete loss of the receptor was further validated through flow cytometry.

#### Magnetic circRNA preparation

CicRNA was covalently labeled at free amine groups with 30 nm–diameter IONPs with N-succinimidyl ester functionalization (Sigma). CircRNA were pelleted by centrifugation, resuspended in PBS to 1 ug/ul and incubated with 12 mg/ml of IONP for 45 min at room temperature with gentle shaking. Labeled circRNA was then purified using G50 microspin columns to remove residual IONP. After magnet conjugation, circRNA was stored at 4°C for less than 48 hours.

#### Magnetic screen

J774 cells carrying the sgRNA library were plated at 50 million cells per 15 cm dish. Cell medium was exchanged with 15 ml of serum-free growth medium and returned to the incubator for 5 min. Medium was replaced with 15 ml of prewarmed serum-free growth medium containing 10 ug of IONP-circRNA per ml of media, and returned to the incubator. After the 24-hour incubation period, the medium was aspirated, and the plates were gently washed twice with PBS. The cells were detached using a cell scraper, collected by centrifugation, and resuspended in 5 ml of magnetic separation buffer. The cell suspension was then processed for magnetic separation using LS columns according to the manufacturer’s instructions (Miltenyi Biotec). Bound and unbound cell fractions were then pelleted and stored at −80°C for up to 1 week.

#### Screen analysis

Library preparation and screen analysis was performed as previously described^61^. Briefly, genomic DNA was extracted for all each purified population using QIAGEN Blood Maxi Kit. sgRNA sequences were amplified and prepared for sequencing by two sequential PCR reactions as previously described^38^. Products were sequenced using an Illumina NextSeq (30–40 million reads per library). Guide composition and comparisons across bound and unbound fractions were analyzed using casTLE^38^. P values were estimated by permuting gene-targeting guides, and hits were called using FDR thresholds calculated via the Benjamini-Hochberg procedure.

### QUANTIFICATION AND STATISTICAL ANALYSIS

Statistical analysis was performed with Prism (GraphPad Software v9.2.0). For comparing two groups, P values were determined using Student’s t-tests (two-tailed). For comparing more than two groups, one-way ANOVAs followed by Tukey’s test were applied. All of the statistical details of experiments can be found in the figure legends. Differences between groups were considered significant for P values < 0.05. No statistical methods were used to predetermine sample sizes. Data collection and analysis were not performed blind to the conditions of the experiments.

## Supplementary Material

1

2

3

4

## Figures and Tables

**Table:** Key resources table

REAGENT or RESOURCE	SOURCE	IDENTIFIER
Antibodies		
anti-human CD14 Antibody	BioLegend	Cat# 325611; RRID: AB_830684
anti-human CD3 Antibody	BioLegend	Cat# 300422; RRID: AB_493092
anti-human CD19 Antibody	BioLegend	Cat# 302222; RRID: AB_492935
anti-human CD56 Antibody	BioLegend	Cat# 318313; RRID: AB_604095
anti-human CD15 Antibody	BioLegend	Cat# 323012; RRID: AB_756018
anti-human CD41 Antibody	BioLegend	Cat# 303725; RRID: AB_2566536
anti-human CD3 Antibody	BioLegend	Cat# 300405; RRID: AB_314059
anti-mouse/human CD11c Antibody	BioLegend	Cat# 301629; RRID: AB_ 11219609
anti-human HLA-DR Antibody	BioLegend	Cat# 307649; RRID: AB_ 2562544
anti-human CD16 Antibody	BioLegend	Cat# 360733; RRID: AB_ 2800993
anti-human CD123 Antibody	BioLegend	Cat# 306017; RRID: AB_ 10900244
anti-human CD19 Antibody	BioLegend	Cat# 302225; RRID: AB_ 493750
anti-mouse CD8a	BD Biosciences	Cat# 563046
anti-mouse TCR Vα2 Antibody	BioLegend	Cat# 127807; RRID: AB_ 1134184
Biological samples		
Healthy adult buffy coat	Stanford Blood Center	N/A
Chemicals, peptides, and recombinant proteins		
DnaseI	Ambion	Cat# AM2222
CleanCap Reagent AG	TriLink Biotechnologies	Cat# N-7113–5
Fluorescein-12-UTP	Sigma-Aldrich	Cat# 11427857910
N6-Methyladenosine-5′-Triphosphate	TriLink Biotechnologies	Cat# N-1013
2’-O-Methylcytidine-5’-Triphosphate	TriLink Biotechnologies	Cat# N-1016–1
RNaseR	MCLAB	Cat# RNASR-200
Dapi Solution	BD Biosciences	Cat# 564907
Human TruStain FcX	BioLegend	Cat# 422301
Zombie NIR Fixable Viability	BioLegend	Cat# 423105
Poly(I:C)	Sigma-Aldrich	Cat# P9582
tRNA	Sigma-Aldrich	TRNABAK-RO
Heparin sodium salt	Sigma-Aldrich	Cat# H3149
Sodium azide	Sigma-Aldrich	Cat# S2002
2-Deoxy-D-glucose	Tocris	Cat# 4515
Oligomycin	Sigma-Aldrich	Cat# O4876
LPS	Sigma-Aldrich	Cat# L4516
PMA	Sigma-Aldrich	Cat# P1585
OVA257–264	Sigma-Aldrich	Cat# S7951
Ovalbumin	InvivoGen	Cat# vac-pova
CpG	InvivoGen	Cat# ODN 1585
Iron oxide(II,III) magnetic	Sigma-Aldrich	7 Cat# 47467–1G
Fucoidan	Sigma-Aldrich	Cat# F8190–500MG
D-(+)-Galactose	Sigma-Aldrich	Cat# G0750–10G
TAK-242	Tocris	Cat# 6587
BC-LI-0186	Tocris	Cat# 6791
Cytochalasin D	Tocris	Cat# 1233
Imipramine hydrochloride	Sigma-Aldrich	Cat# I7379–5G
Phenoxybenzamine hydrochloride	Sigma-Aldrich	Cat# B019–250MG
Fluorescein isothiocyanate-dextran	Sigma-Aldrich	Cat# FD4–100MG
Transferrin From Human Serum, Alexa Fluor 488 Conjugate	Invitrogen	Cat# T13342
Deposited data		
RNA-seq raw data	This paper	GEO: GSE264160
CRISPR screen raw data	This paper	GEO: GSE264160
Experimental models: Cell lines		
Mouse: RAW264.7	ATCC	Cat# TIB-71
Mouse: J774-Cas9	Bassik Laboratory	N/A
Mouse: MutuDC	ABM	Cat# T0528
*Human: U937-Cas9*	Bassik Laboratory	N/A
Human: THP1	ATCC	Cat# TIB-202
Human: KG-1	ATCC	Cat# CCL-246
Human: Calu-3	ATCC	Cat# HTB-55
Human: IMR-90	ATCC	Cat# CCL-186
Human: Hep G2	ATCC	Cat# HB-8065
Critical commercial assays		
Mycoplasma kit	Lonza	Cat# LT07–318
HiScribe T7 High Yield RNA Synthesis Kit	NEB	Cat# E2040S
DNA Clean & Concentrator	Zymo Research	Cat# D4030
High Sensitivity RNA ScreenTape Analysis	Agilent	Cat# 5067–5576
Label IT Nucleic Acid Labeling Kit, Fluorescein	Mirus Bio	Cat# MIR 3200
Fixable Viability Kit	BioLegend	Cat# 423109
EasySep Human Monocyte Enrichment Kit without CD16 Depletion	STEMCELL Technologies	Cat#19059
TransIT-mRNA transfection kit	Mirus Bio	Cat# MIR 2225
Nano-Glo Dual-Luciferase Reporter Assay	Promega	Cat# N142A
EasySep Mouse Naïve CD8+ T Cell Isolation Kit	STEMCELL Technologies	Cat# 19858
RNeasy Mini Kit	Qiagen	Cat# 74104
Ribo-Zero Plus rRNA Depletion Kit	Illumina	Cat# 20037135
QIAamp DNA Blood Maxi Kit	Qiagen	Cat# 51194
CellTrace CFSE Cell Proliferation Kit	Thermo Fisher	Cat# C34554
Recombinant DNA		
circFOR	Chen et al.^54^	N/A
circNanoluc	Chen et al.^32^	N/A
circOVA	Amaya et al.^15^	N/A
Bassik Lab Mouse CRISPR Knockout Library	Morgens et al.^55^	https://www.addgene.org/pooled-library/bassik-mouse-crispr-knockout/
Software and algorithms		
Prism	GraphPad	https://www.graphpad.com/features
Salmon	Rob Patro et al.^56^	N/A
DESeq2	Love et al.^57^	N/A
ClusterProfiler	Guangchuang et al.^58^	N/A
casTLE	Morgens et al.^38^	N/A

## Highlights:

- Extracellular circRNA is internalized by select immune cells in an active process.
- CircRNA uptake can lead to translation and antigen presentation.
- CircRNA internalization induces macrophage activation and distinct gene expression.
- CRISPR screen identifies MSR1 receptor mediated phagocytosis and macropinocytosis.

## References

[1] Jeck WR, Sorrentino JA, Wang K, Slevin MK, Burd CE, Liu J, Marzluff WF, and Sharpless NE (2013). Circular RNAs are abundant, conserved, and associated with ALU repeats. RNA 19, 141–157. 23249747 10.1261/rna.035667.112 PMC3543092

[2] Hansen TB, Jensen TI, Clausen BH, Bramsen JB, Finsen B, Damgaard CK, and Kjems J. (2013). Natural RNA circles function as efficient microRNA sponges. Nature 495, 384–388. 23446346 10.1038/nature11993

[3] Ashwal-Fluss R, Meyer M, Pamudurti NR, Ivanov A, Bartok O, Hanan M, Evantal N, Memczak S, Rajewsky N, and Kadener S. (2014). circRNA Biogenesis Competes with PremRNA Splicing. Mol. Cell 56, 55–66. 25242144 10.1016/j.molcel.2014.08.019

[4] Holdt LM, Stahringer A, Sass K, Pichler G, Kulak NA, Wilfert W, Kohlmaier A, Herbst A, Northoff BH, Nicolaou A, (2016). Circular non-coding RNA ANRIL modulates ribosomal RNA maturation and atherosclerosis in humans. Nat. Commun. 7, 12429. 27539542 10.1038/ncomms12429 PMC4992165

[5] Yang Y, Fan X, Mao M, Song X, Wu P, Zhang Y, Jin Y, Yang Y, Chen L-L, Wang Y, (2017). Extensive translation of circular RNAs driven by N6-methyladenosine. Cell Res. 27, 626–641. 28281539 10.1038/cr.2017.31 PMC5520850

[6] Verduci L, Tarcitano E, Strano S, Yarden Y, and Blandino G. (2021). CircRNAs: role in human diseases and potential use as biomarkers. Cell Death Dis. 12, 468. 33976116 10.1038/s41419-021-03743-3 PMC8113373

[7] Memczak S, Papavasileiou P, Peters O, and Rajewsky N. (2015). Identification and Characterization of Circular RNAs As a New Class of Putative Biomarkers in Human Blood. PLoS ONE 10, e0141214. 10.1371/journal.pone.0141214 PMC4617279 26485708

[8] Koh W, Pan W, Gawad C, Fan HC, Kerchner GA, Wyss-Coray T, Blumenfeld YJ, El-Sayed YY, and Quake SR (2014). Noninvasive in vivo monitoring of tissue-specific global gene expression in humans. Proc. Natl. Acad. Sci. 111, 7361–7366. 24799715 10.1073/pnas.1405528111 PMC4034220

[9] Bahn JH, Zhang Q, Li F, Chan T-M, Lin X, Kim Y, Wong DTW, and Xiao X. (2014). The landscape of microRNA, Piwi-interacting RNA, and circular RNA in human saliva. Clin. Chem. 61, 221–230. 25376581 10.1373/clinchem.2014.230433 PMC4332885

[10] Shi X, Wang B, Feng X, Xu Y, Lu K, and Sun M. (2020). circRNAs and Exosomes: A Mysterious Frontier for Human Cancer. Mol. Ther. - Nucleic Acids 19, 384–392. 31887549 10.1016/j.omtn.2019.11.023 PMC6939016

[11] Abbas MN, Kausar S, Gul I, Li J, Yu H, Dong M, and Cui H. (2023). The Potential Biological Roles of Circular RNAs in the Immune Systems of Insects to Pathogen Invasion. Genes 14, 895. 37107653 10.3390/genes14040895 PMC10137924

[12] Li X, Liu C-X, Xue W, Zhang Y, Jiang S, Yin Q-F, Wei J, Yao R-W, Yang L, and Chen L-L (2017). Coordinated circRNA Biogenesis and Function with NF90/NF110 in Viral Infection. Mol. Cell 67, 214–227.e7. 28625552 10.1016/j.molcel.2017.05.023

[13] Chen YG, Chen R, Ahmad S, Verma R, Kasturi SP, Amaya L, Broughton JP, Kim J, Cadena C, Pulendran B, (2019). N6-Methyladenosine Modification Controls Circular RNA Immunity. Mol. Cell 76, 96–109.e9. 31474572 10.1016/j.molcel.2019.07.016 PMC6778039

[14] Qu L, Yi Z, Shen Y, Lin L, Chen F, Xu Y, Wu Z, Tang H, Zhang X, Tian F, (2022). Circular RNA vaccines against SARS-CoV-2 and emerging variants. Cell 185, 1728–1744.e16. 10.1016/j.cell.2022.03.044 PMC8971115 35460644

[15] Amaya L, Grigoryan L, Li Z, Lee A, Wender PA, Pulendran B, and Chang HY (2023). Circular RNA vaccine induces potent T cell responses. Proc. Natl. Acad. Sci. 120, e2302191120. 10.1073/pnas.2302191120 PMC10193964 37155869

[16] Chen YG, Kim MV, Chen X, Batista PJ, Aoyama S, Wilusz JE, Iwasaki A, and Chang HY (2017). Sensing Self and Foreign Circular RNAs by Intron Identity. Mol. Cell 67, 228–238.e5. 28625551 10.1016/j.molcel.2017.05.022 PMC5610545

[17] Wolff JA, Malone RW, Williams P, Chong W, Acsadi G, Jani A, and Felgner PL (1990). Direct Gene Transfer into Mouse Muscle in Vivo. Science 247, 1465–1468. 1690918 10.1126/science.1690918

[18] Foroozandeh P, and Aziz AA (2018). Insight into Cellular Uptake and Intracellular Trafficking of Nanoparticles. Nanoscale Res. Lett. 13, 339. 30361809 10.1186/s11671-018-2728-6 PMC6202307

[19] Vlassov VV, Blakireva LA, and Yakubov LA (1994). Transport of oligonucleotides across natural and model membranes. Biochim. Biophys. Acta (BBA) - Rev. Biomembr. 1197, 95–108. 10.1016/0304-4157(94)90001-9 8031827

[20] Lewis DL, Hagstrom JE, Loomis AG, Wolff JA, and Herweijer H. (2002). Efficient delivery of siRNA for inhibition of gene expression in postnatal mice. Nat. Genet. 32, 107–108. 12145662 10.1038/ng944

[21] Ulvila J, Parikka M, Kleino A, Sormunen R, Ezekowitz RA, Kocks C, and Rämet M. (2006). Double-stranded RNA Is Internalized by Scavenger Receptor-mediated Endocytosis in Drosophila S2 Cells*. J. Biol. Chem. 281, 14370–14375. 16531407 10.1074/jbc.M513868200

[22] Diken M, Kreiter S, Selmi A, Britten CM, Huber C, Türeci Ö, and Sahin U. (2011). Selective uptake of naked vaccine RNA by dendritic cells is driven by macropinocytosis and abrogated upon DC maturation. Gene Ther. 18, 702–708. 21368901 10.1038/gt.2011.17

[23] Tatematsu M, Funami K, Seya T, and Matsumoto M. (2018). Extracellular RNA Sensing by Pattern Recognition Receptors. J. Innate Immun. 10, 398–406. 30404092 10.1159/000494034 PMC6784046

[24] Kreiter S, Selmi A, Diken M, Koslowski M, Britten CM, Huber C, Türeci Ö, and Sahin U. (2010). Intranodal Vaccination with Naked Antigen-Encoding RNA Elicits Potent Prophylactic and Therapeutic Antitumoral Immunity. Cancer Res. 70, 9031–9040. 21045153 10.1158/0008-5472.CAN-10-0699

[25] Takakura Y, Takagi T, Hashiguchi M, Nishikawa M, Yamashita F, Doi T, Imanishi T, Suzuki H, Kodama T, and Hashida M. (1999). Characterization of Plasmid DNA Binding and Uptake by Peritoneal Macrophages from Class A Scavenger Receptor Knockout Mice. Pharm. Res. 16, 503–508. 10227703 10.1023/a:1018842210588

[26] Granstein RD, Ding W, and Ozawa H. (2000). Induction of Anti-Tumor Immunity with Epidermal Cells Pulsed with Tumor-Derived RNA or Intradermal Administration of RNA. J. Investig. Dermatol. 114, 632–636. 10733665 10.1046/j.1523-1747.2000.00929.x

[27] Carralot J-P, Probst J, Hoerr I, Scheel B, Teufel R, Jung G, Rammensee H-G, and Pascolo S. (2004). Polarization of immunity induced by direct injection of naked sequence-stabilized mRNA vaccines. Cell. Mol. Life Sci. CMLS 61, 2418–2424. 15378210 10.1007/s00018-004-4255-0 PMC7079797

[28] Weide B, Carralot J-P, Reese A, Scheel B, Eigentler , Hoerr I, Rammensee H-G, Garbe C, and Pascolo S. (2008). Results of the First Phase I&sol;II Clinical Vaccination Trial With Direct Injection of mRNA. J. Immunother. 31, 180–188. 18481387 10.1097/CJI.0b013e31815ce501

[29] Gilleron J, Querbes W, Zeigerer A, Borodovsky A, Marsico G, Schubert U, Manygoats K, Seifert S, Andree C, Stöter M, (2013). Image-based analysis of lipid nanoparticle–mediated siRNA delivery, intracellular trafficking and endosomal escape. Nat. Biotechnol. 31, 638–646. 23792630 10.1038/nbt.2612

[30] Alinovskaya LI, Sedykh SE, Ivanisenko NV, Soboleva SE, and Nevinsky GA (2018). How human serum albumin recognizes DNA and RNA. Biol. Chem. 399, 347–360. 29252186 10.1515/hsz-2017-0243

[31] Akinc A, Querbes W, De S, Qin J, Frank-Kamenetsky M, Jayaprakash KN, Jayaraman M, Rajeev KG, Cantley WL, Dorkin JR, (2010). Targeted Delivery of RNAi Therapeutics With Endogenous and Exogenous Ligand-Based Mechanisms. Mol. Ther. 18, 1357–1364. 20461061 10.1038/mt.2010.85 PMC2911264

[32] Chen R, Wang SK, Belk JA, Amaya L, Li Z, Cardenas A, Abe BT, Chen C-K, Wender PA, and Chang HY (2023). Engineering circular RNA for enhanced protein production. Nat. Biotechnol. 41, 262–272. 35851375 10.1038/s41587-022-01393-0 PMC9931579

[33] Askew D, Chu RS, Krieg AM, and Harding CV (2000). CpG DNA Induces Maturation of Dendritic Cells with Distinct Effects on Nascent and Recycling MHC-II Antigen-Processing Mechanisms. J. Immunol. 165, 6889–6895. 11120813 10.4049/jimmunol.165.12.6889

[34] Rijkers GT, Weterings N, Obregon-Henao A, Lepolder M, Dutt TS, Overveld FJ van, and Henao-Tamayo M. (2021). Antigen Presentation of mRNA-Based and Virus-Vectored SARS-CoV-2 Vaccines. Vaccines 9, 848. 34451973 10.3390/vaccines9080848 PMC8402319

[35] Fiszer-Kierzkowska A, Vydra N, Wysocka-Wycisk A, Kronekova Z, Jarząb M, Lisowska KM, and Krawczyk Z. (2011). Liposome-based DNA carriers may induce cellular stress response and change gene expression pattern in transfected cells. BMC Mol. Biol. 12, 27–27. 21663599 10.1186/1471-2199-12-27 PMC3132718

[36] Liu Z, and Roche PA (2015). Macropinocytosis in phagocytes: regulation of MHC class-II-restricted antigen presentation in dendritic cells. Front. Physiol. 6, 1. 25688210 10.3389/fphys.2015.00001 PMC4311620

[37] Elguindy MM, Kopp F, Goodarzi M, Rehfeld F, Thomas A, Chang T-C, and Mendell JT (2019). PUMILIO, but not RBMX, binding is required for regulation of genomic stability by noncoding RNA NORAD. eLife 8, e48625. 10.7554/eLife.48625 PMC6677556 31343408

[38] Morgens DW, Deans RM, Li A, and Bassik MC (2016). Systematic comparison of CRISPR/Cas9 and RNAi screens for essential genes. Nat. Biotechnol. 34, 634–636. 27159373 10.1038/nbt.3567 PMC4900911

[39] Rougerie P, Miskolci V, and Cox D. (2013). Generation of membrane structures during phagocytosis and chemotaxis of macrophages: role and regulation of the actin cytoskeleton. Immunol. Rev. 256, 222–239. 24117824 10.1111/imr.12118 PMC3806206

[40] Zhang W, He T, Wang Q, Li X, Wei J, Hou X, Zhang B, Huang L, and Wang L. (2014). Interleukin-1 Receptor-associated Kinase-2 Genetic Variant rs708035 Increases NF-κB Activity through Promoting TRAF6 Ubiquitination*. J. Biol. Chem. 289, 12507–12519. 24662294 10.1074/jbc.M113.538009 PMC4007444

[41] Gudgeon J, Marín-Rubio JL, and Trost M. (2022). The role of macrophage scavenger receptor 1 (MSR1) in inflammatory disorders and cancer. Front. Immunol. 13, 1012002. 10.3389/fimmu.2022.1012002 PMC9618966 36325338

[42] Mietus-Snyder M, Glass CK, and Pitas RE (1998). Transcriptional Activation of Scavenger Receptor Expression in Human Smooth Muscle Cells Requires AP-1/c-Jun and C/EBPβ. Arter., Thromb., Vasc. Biol. 18, 1440–1449. 10.1161/01.atv.18.9.1440 9743233

[43] Taban Q, Mumtaz PT, Masoodi KZ, Haq E, and Ahmad SM (2022). Scavenger receptors in host defense: from functional aspects to mode of action. Cell Commun. Signal. : CCS 20, 2. 34980167 10.1186/s12964-021-00812-0 PMC8721182

[44] Amiel E, Alonso A, Uematsu S, Akira S, Poynter ME, and Berwin B. (2009). Pivotal Advance: Toll-like receptor regulation of scavenger receptor-A-mediated phagocytosis. J. Leukoc. Biol. 85, 595–605. 19112093 10.1189/jlb.1008631 PMC2656429

[45] Taub N, Nairz M, Hilber D, Hess MW, Weiss G, and Huber LA (2012). The late endosomal adaptor p14 is a macrophage host-defense factor against Salmonella infection. J. Cell Sci. 125, 2698–2708. 22427693 10.1242/jcs.100073

[46] Scheffler JM, Sparber F, Tripp CH, Herrmann C, Humenberger A, Blitz J, Romani N, Stoitzner P, and Huber LA (2014). LAMTOR2 regulates dendritic cell homeostasis through FLT3-dependent mTOR signalling. Nat. Commun. 5, 5138. 25336251 10.1038/ncomms6138 PMC4220488

[47] Liu H, Zhu L, Dudiki T, Gabanic B, Good L, Podrez EA, Cherepanova OA, Qin J, and Byzova TV (2020). Macrophage Migration and Phagocytosis Are Controlled by Kindlin-3’s Link to the Cytoskeleton. J. Immunol. 204, 1954–1967. 32094207 10.4049/jimmunol.1901134 PMC8203317

[48] Rennick JJ, Johnston APR, and Parton RG (2021). Key principles and methods for studying the endocytosis of biological and nanoparticle therapeutics. Nat. Nanotechnol. 16, 266–276. 33712737 10.1038/s41565-021-00858-8

[49] Lin H, Singla B, Ghoshal P, Faulkner JL, Cherian-Shaw M, O’Connor PM, She J, Chantemele E.J.B. de, and Csányi G. (2018). Identification of novel macropinocytosis inhibitors using a rational screen of Food and Drug Administration-approved drugs. Br. J. Pharmacol. 175, 3640–3655. 29953580 10.1111/bph.14429 PMC6109223

[50] Varkouhi AK, Scholte M, Storm G, and Haisma HJ (2011). Endosomal escape pathways for delivery of biologicals. J. Control. Release 151, 220–228. 21078351 10.1016/j.jconrel.2010.11.004

[51] Bus T, Traeger A, and Schubert US (2018). The great escape: how cationic polyplexes overcome the endosomal barrier. J. Mater. Chem. B 6, 6904–6918. 32254575 10.1039/c8tb00967h

[52] Unworth H, Raguz S, Edwards HJ, Higgins CF, and Yagüe E. (2010). mRNA escape from stress granule sequestration is dictated by localization to the endoplasmic reticulum. FASEB J. 24, 3370–3380. 20453113 10.1096/fj.09-151142

[53] Zhang Z, Yang T, and Xiao J. (2018). Circular RNAs: Promising Biomarkers for Human Diseases. EBioMedicine 34, 267–274. 30078734 10.1016/j.ebiom.2018.07.036 PMC6116471

[54] Chen YG, Satpathy AT, and Chang HY (2017). Gene regulation in the immune system by long noncoding RNAs. Nat. Immunol. 18, 962–972. 28829444 10.1038/ni.3771 PMC9830650

[55] Morgens DW, Wainberg M, Boyle EA, Ursu O, Araya CL, Tsui CK, Haney MS, Hess GT, Han K, Jeng EE, (2017). Genome-scale measurement of off-target activity using Cas9 toxicity in high-throughput screens. Nat. Commun. 8, 15178. 28474669 10.1038/ncomms15178 PMC5424143

[56] Patro R, Duggal G, Love MI, Irizarry RA, and Kingsford C. (2017). Salmon provides fast and bias-aware quantification of transcript expression. Nat. Methods 14, 417–419. 28263959 10.1038/nmeth.4197 PMC5600148

[57] Love MI, Huber W, and Anders S. (2014). Moderated estimation of fold change and dispersion for RNA-seq data with DESeq2. Genome Biol. 15, 550. 25516281 10.1186/s13059-014-0550-8 PMC4302049

[58] Yu G, Wang L-G, Han Y, and He Q-Y (2012). clusterProfiler: an R Package for Comparing Biological Themes Among Gene Clusters. OMICS: A J. Integr. Biol. 16, 284–287. 10.1089/omi.2011.0118 PMC3339379 22455463

[59] Liu J, Xavy S, Mihardja S, Chen S, Sompalli K, Feng D, Choi T, Agoram B, Majeti R, Weissman IL, (2019). Targeting macrophage checkpoint inhibitor SIRPα for anticancer therapy. JCI insight 5. 10.1172/jci.insight.134728 PMC7406266 32427583

[60] Kamber RA, Nishiga Y, Morton B, Banuelos AM, Barkal AA, Vences-Catalán F, Gu M, Fernandez D, Seoane JA, Yao D, (2021). Inter-cellular CRISPR screens reveal regulators of cancer cell phagocytosis. Nature 597, 549–554. 34497417 10.1038/s41586-021-03879-4 PMC9419706

[61] Haney MS, Bohlen CJ, Morgens DW, Ousey JA, Barkal AA, Tsui CK, Ego BK, Levin R, Kamber RA, Collins H, (2018). Identification of phagocytosis regulators using magnetic genome-wide CRISPR screens. Nat. Genet. 50, 1716–1727. 30397336 10.1038/s41588-018-0254-1 PMC6719718

