# Supplementary material for: Pathways for macrophage uptake of cell-free circular RNAs
Source: Mol Cell. Author manuscript; Available in PMC 2024 Jul 2. (PMC11218042; doi:10.1016/j.molcel.2024.04.022)
Supplement: 1 [file NIHMS1992517-supplement-1.pdf]

Supplemental Information

Supplementary Figures

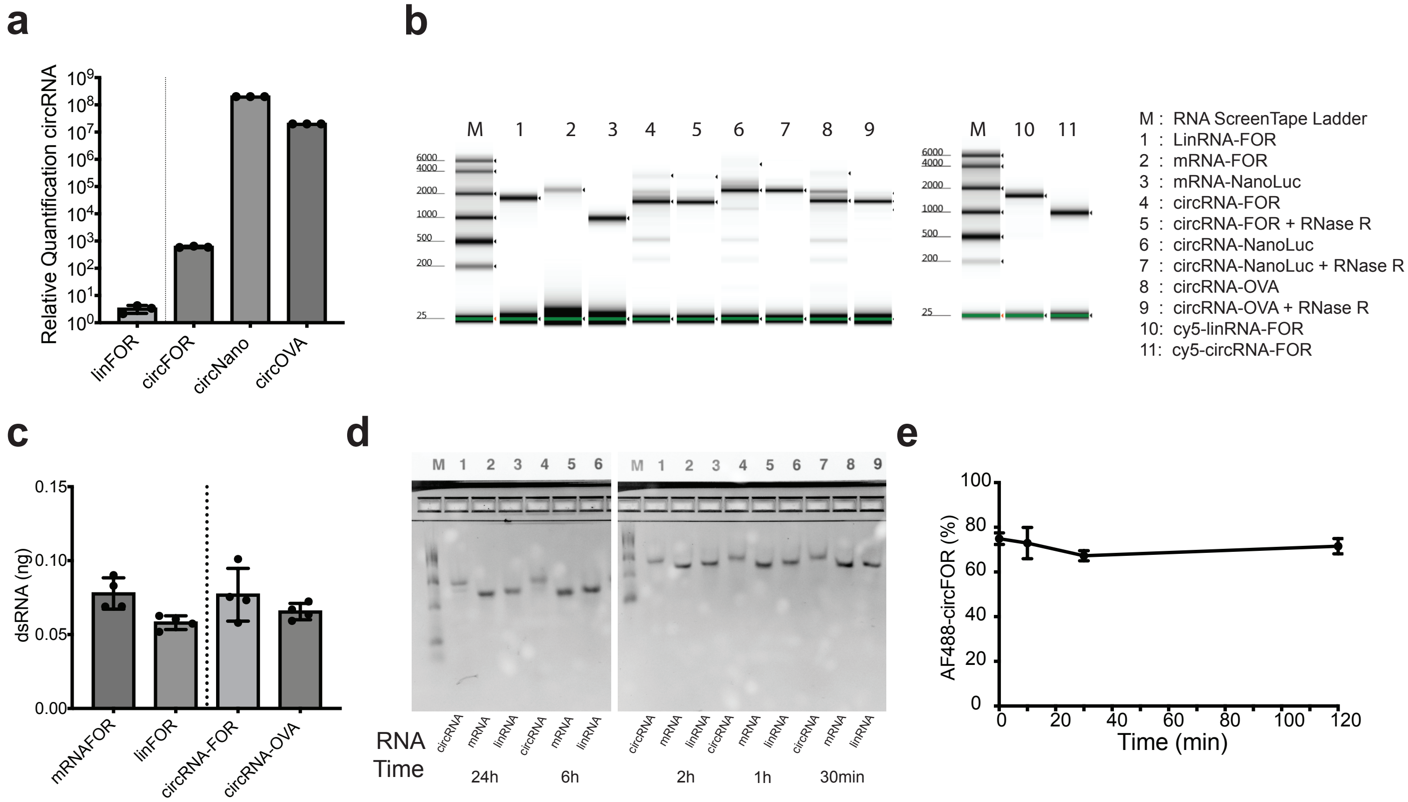

Supplementary Figure. 1. Quality assessment of in vitro transcribed circRNA. Related to STAR Methods. **a,** Relative quantification of circular RNA (circRNA) by qPCR using divergent primers that span the splice junction and only amplify products when circRNA is present. **b,** Quality control of RNA in vitro transcription and assessment of circRNA purity after RNase R digestion with Tape Station. **c,** Quantitative ELISA for sensitive and selective detection of double-stranded RNA (dsRNA) molecules larger than 30-40 base pairs. **d,** Gel electrophoresis analysis of RNA samples after incubation in serum-free media for durations ranging from 30 minutes to 24 hours. **e,** Percentage of circRNA uptake after incubation in serum-free medium for durations ranging from 10 minutes to 2 hours.

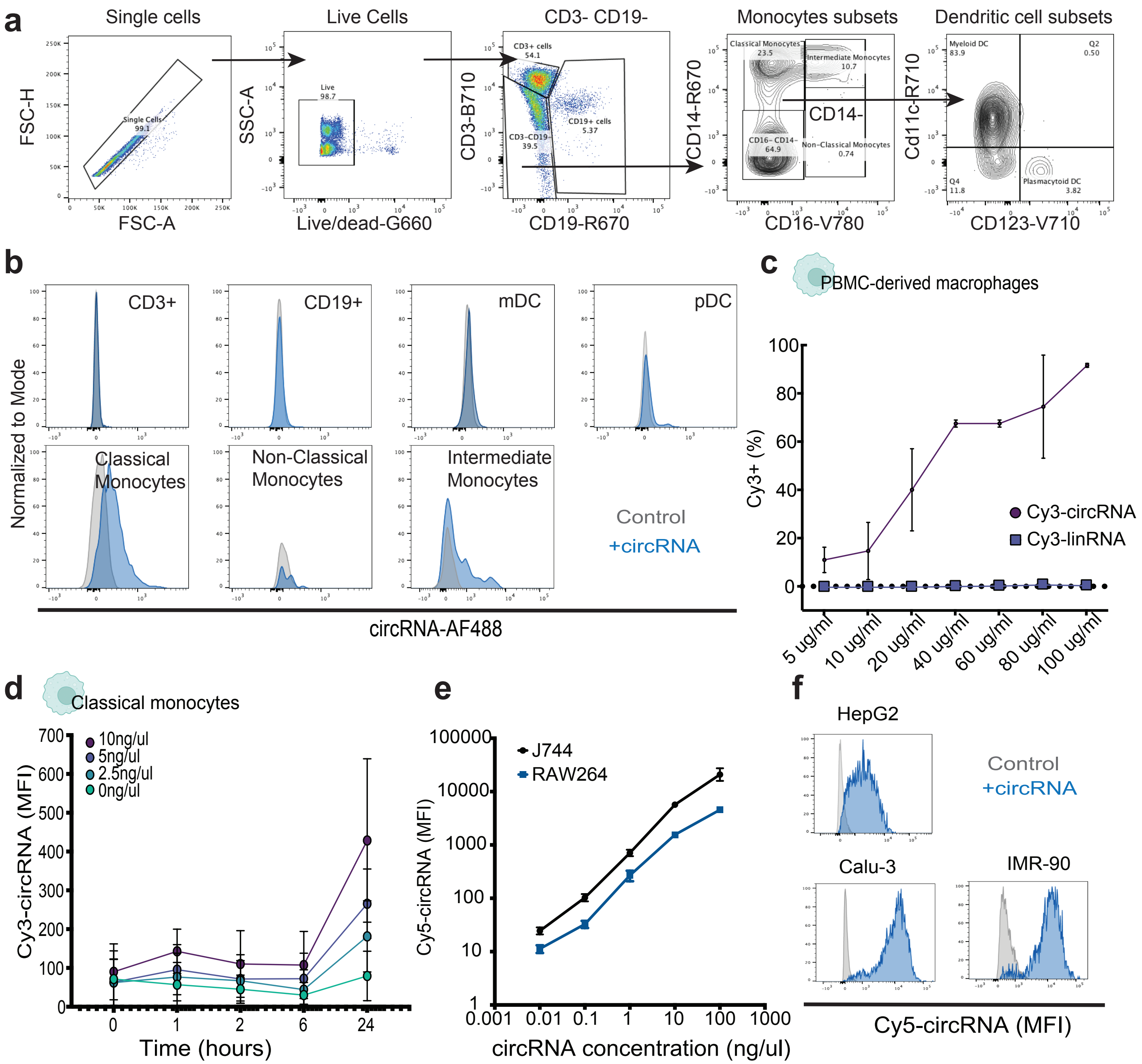

Supplementary Figure. 2. Extracellular circular RNA uptake is cell specific. Related to Figure 1. **a,** Gating strategy used to define myeloid human cell population after uptake of circRNA. **b,** Histogram representation of fluorescent intensity shift after circRNA uptake in human immune cell subsets corresponding to **Fig. 1c** (representative sample). **c,** Flow cytometry quantification of increasing concentration of circRNA and linRNA in human PBMC-derived macrophages. **d,** CircRNA is taken up by human classical monocytes in a dose and time dependent manner (n = 4, bars represent SEM). **e,** circRNA is taken up by J774 and RAW cells in a dose dependent manner (n = 4, bars represent SEM). **f,** Histogram representation of fluorescence intensity shift after circRNA uptake in human liver and lung cell lines (representative sample).

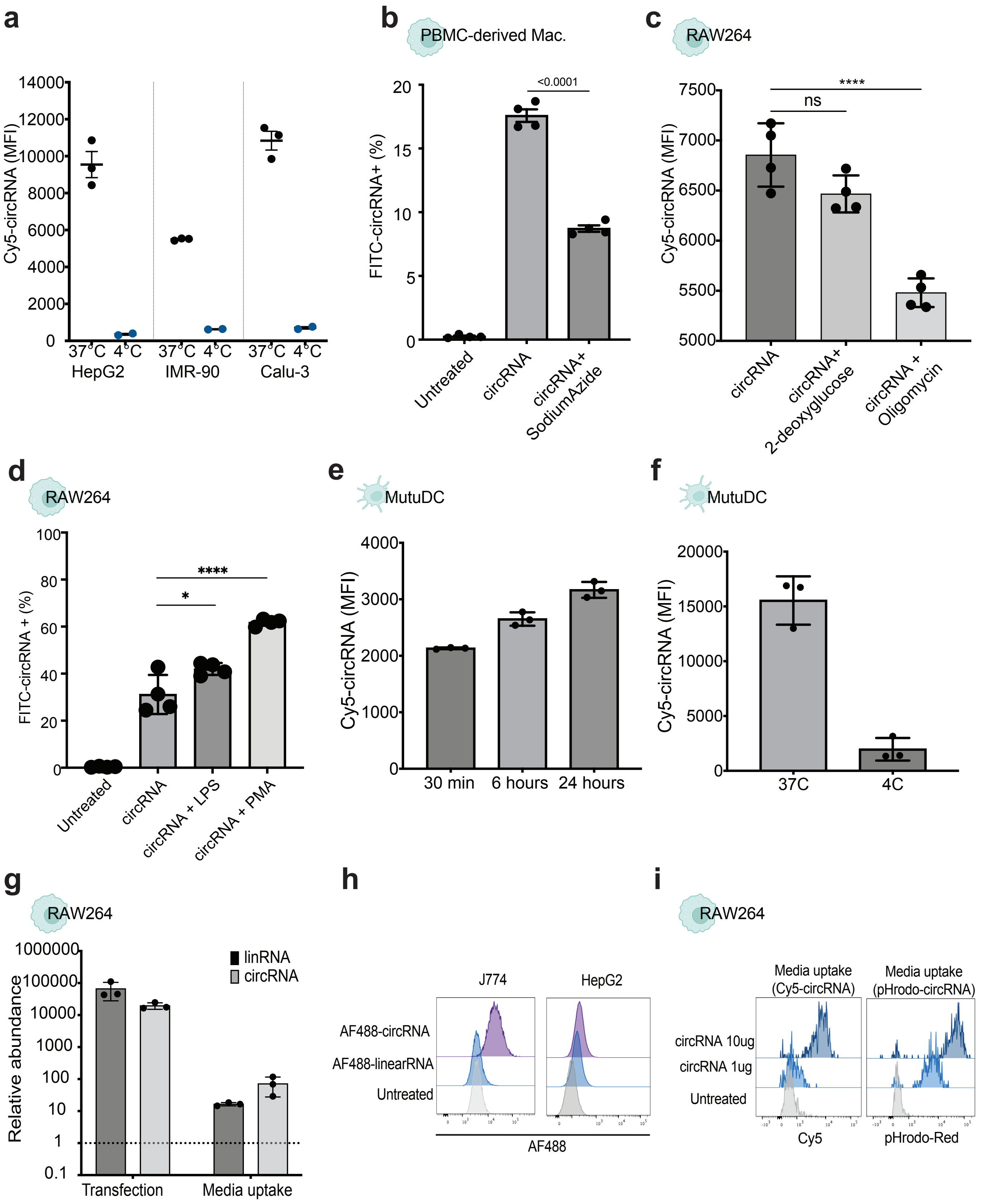

Supplementary Figure 3. CircRNA uptake is a fast and active process in distinct cell lines. Related to Figure 2. **a,** circRNA uptake is inhibited at cold temperatures in human liver and lung cell lines (n = 3, bars represent SEM). **b,** Sodium azide effect on circRNA uptake in RAW264 cells (n = 3, bars represent SEM). **c,** Distinct metabolic inhibitors negatively inhibit circRNA uptake in RAW264 cells (n = 4, bars represent SEM). **d,** Maturation signals promote circRNA uptake in RAW264 cells (n = 3, bars represent SEM). **e,** Time course analysis of circRNA uptake measured by flow cytometry in MutuDC cells (n = 3, bars represent SEM). **f,**

Temperature effect on circRNA uptake in MutuDC cells (n = 3, bars represent SEM). **g,** qRT-PCR quantification of circRNA compared to mRNA after uptake or transfection in RAW264 cells (n = 3, bars represent SEM). **h,** Representative sample of circRNA uptake compared to mRNA in distinct cell types measured by flow cytometry. **i,** Representative sample of circRNA uptake measurement comparison between cy5-circRNA or pHrodo-circRNA at distinct concentrations in RAW264 cells. One-way ANOVA followed by Tukey's test was applied in b-d. *P < 0.05, ****P < 0.0001. ns = not significant.

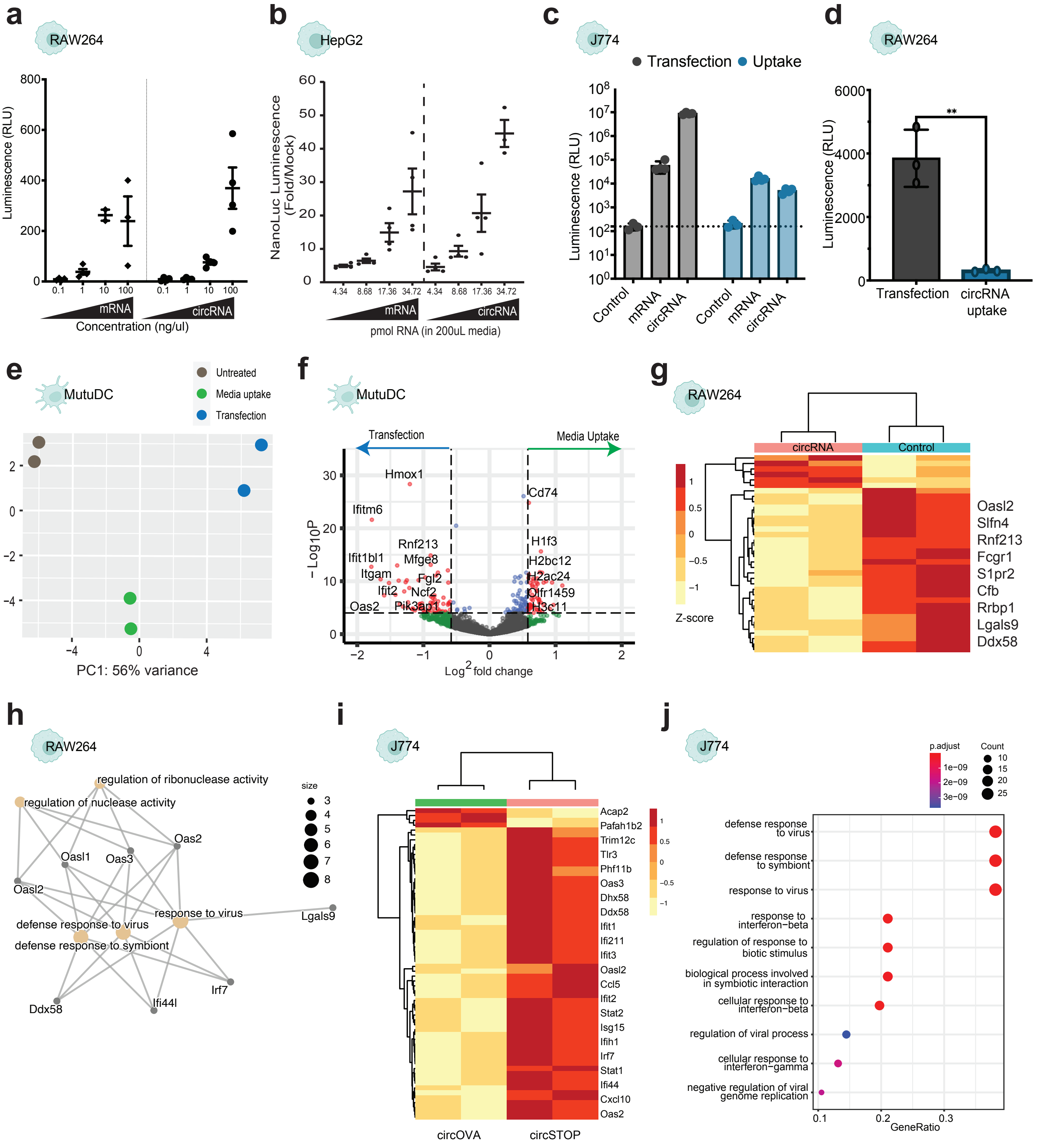

Supplementary Figure 4. CircRNA uptake compared to cell transfection. Related to Figure 3. Nanoluciferase intensity measured in **a,** RAW and **b,** HepG2 cells shows similar translation efficiency after uptake of mRNA (left) or circRNA (right) encoding Nanoluciferase protein. **c,** Nanoluciferase intensity measured in J774 cells after transfection and uptake of circRNA and mRNA compared to untranslatable control (n = 3, bars represent SEM). **d,** Nanoluciferase intensity measured in RAW264 cells after transfection and uptake (n = 3, bars represent SEM). Statistical significance was calculated using a two-tailed unpaired t-test, *P < 0.05. **e,** PCA analysis of normalized and transformed transcriptome counts grouped by condition: Untreated, circRNA and CART-circRNA (n=2). **f,**

Volcano plot with the log2 fold changes in gene expression between circRNA uptake and circRNA transfection. The gene symbols of the top differentiated genes ordered by padj value are displayed. **g**, Heatmap of normalized expression data showing differentially regulated genes following circRNA uptake in RAW264 cells **h**, Functional analysis of the top differentially expressed genes and their linkages with biological concepts (GO terms) after circRNA uptake in RAW264 cells. **i**, Heatmap of normalized expression data showing differentially regulated genes following circRNA uptake in J774 cells **j**, Functional analysis of the top differentially expressed genes and their linkages with biological concepts (GO terms) after circRNA uptake in J774 cells.

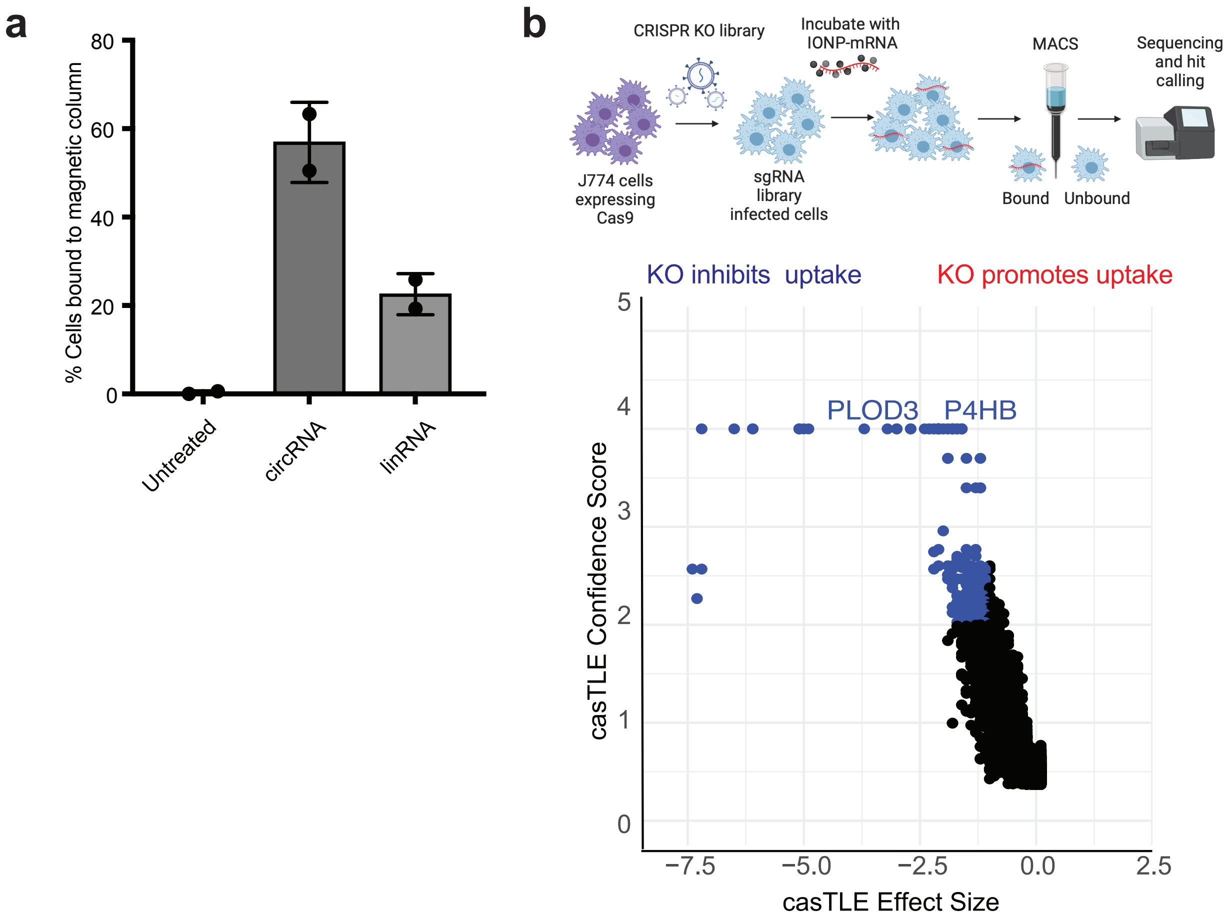

Supplementary Figure 5. CRISPR KO screen for circRNA and linear RNA uptake. Related to Figure 4. **a**, Percentage of J774 cells bound to magnet when IONP is covalently bound to circRNA or mRNA. **b**, Schematic representation of linear mRNA uptake screening using magnetic separation (top) and volcano plot of all genes indicating effect and confidence scores for genome-wide linRNA uptake screen. Effect and confidence scores determined by casTLE. (bottom).

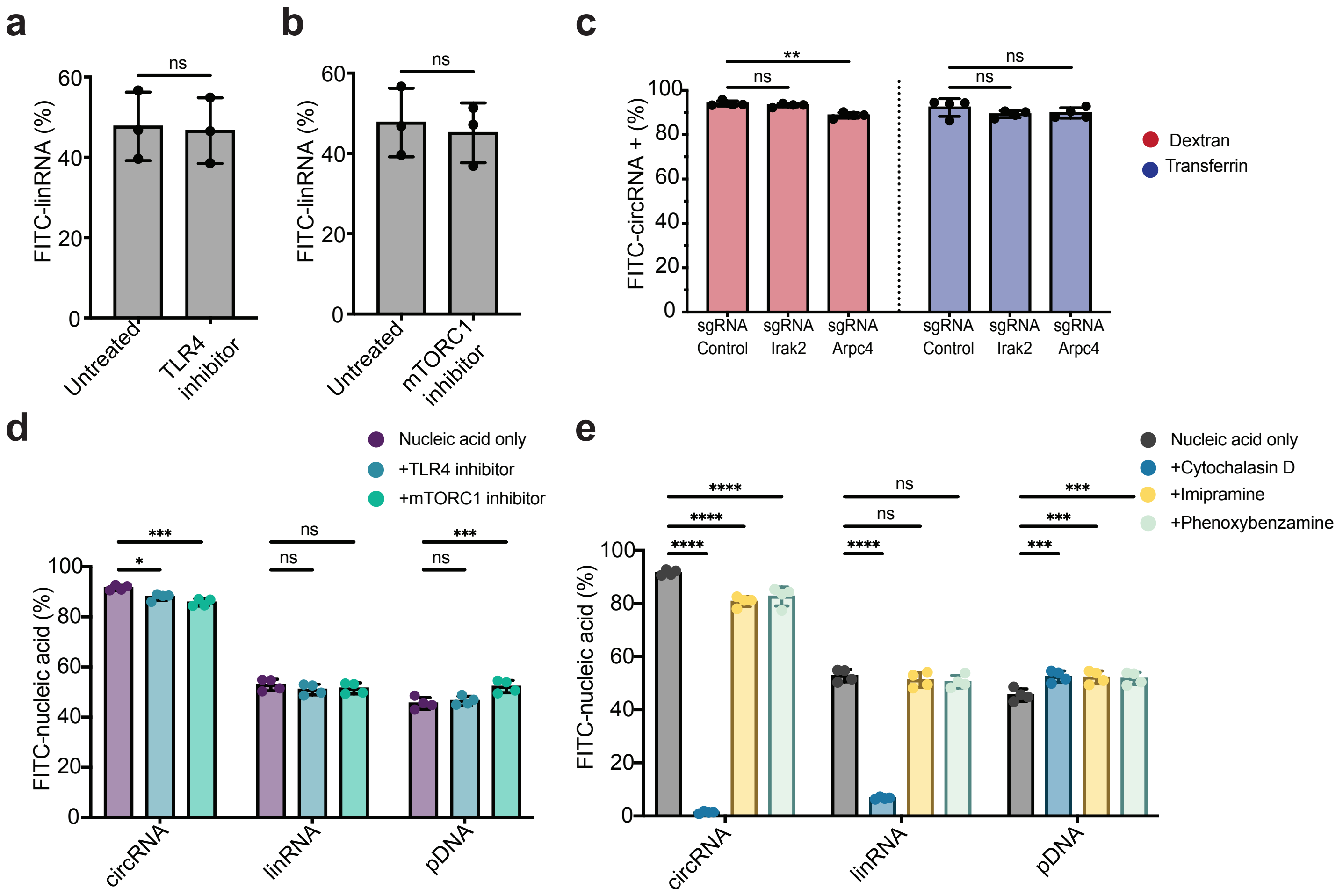

Supplementary Figure 6. Validation of uptake regulators in distinct types of nucleic acids. Related to Figure 5. **a**, Chemical inhibition of TLR4 and its effect in linRNA uptake in J774 cells (n = 3, bars represent SD). **b**, Chemical inhibition of mTORC1 and its effect in linRNA uptake in J774 (n = 3, bars represent SD). Statistical significance in a and b was calculated using Mann-Whitney test. **c,** Cellular uptake of dextran and transferrin after single gene KOs in J774-Cas9 (n = 3, bars represent SD). **d**, Chemical inhibition of TLR4 and mTORC1 and its effect on circular RNA (circRNA), linear RNA (linRNA), and plasmid DNA (pDNA) uptake in J774 cells (n = 3, bars represent SD). **e**, Chemical inhibition of macropinocytosis and its effect on the uptake of circular RNA (circRNA), linear RNA (linRNA), and plasmid DNA (pDNA) in J774 cells (n = 3, bars represent SD). Statistical significance in c-e was calculated using two-way ANOVA and Dunnett's multiple comparisons test. *$P < 0.05$, **$P < 0.01$, ***$P < 0.001$, ****$P < 0.0001$. ns = not significant.

Supplementary Tables

Table S1. List of RNAs used throughout experiments. Related to STAR Methods.

Figure	RNA	CDS	Size (kb)	RNA modification	IRES	5'UTR	3'UTR	Labeling
**Figure 1, 5**	cirRNA	GFP (out of frame)	1.9	none	iCVB3	none	none	Cy3
**Figure 1**	linRNA	GFP (out of frame)	1.9	none	iCVB3	none	none	Cy3
**Figure 1, 2**	cirRNA	GFP (out of frame)	1.9	none	iCVB3	none	none	Cy5
**Figure 2, 3**	cirRNA	GFP (out of frame)	1.9	none	iCVB3	none	none	none
**Figure 2**	linRNA	GFP (out of frame)	1.9	none	iCVB3	none	none	none
**Figure 3, S4**	circOVA	Ovalbumin	2.4	5% 2'-O-MethylIC	synIRES-RC25 (iHRVB3-based)	5′ PABP spacer	HBA1	none
**Figure 4**	cirRNA	GFP (out of frame)	1.9	none	iCVB3	none	none	IONP
**Figure 4**	linRNA	GFP (out of frame)	1.9	none	iCVB3	none	none	FITC
**Figure 4, 5**	cirRNA	GFP (out of frame)	1.9	none	iCVB3	none	none	FITC
**Figure S1, S3, S5**	cirRNA	GFP (out of frame)	1.9	none	iCVB3	none	none	none
**Figure S1, S3, S5**	linRNA	GFP (out of frame)	1.9	none	iCVB3	none	none	none
**Figure S1**	mRNA	GFP (out of frame)	1.9	CleanCap and 100% N1Ψ	iCVB3	none	none	none
**Figure S2**	cirRNA	GFP (out of frame)	1.9	none	iCVB3	none	none	Cy3
**Figure S2**	linRNA	GFP (out of frame)	1.9	none	iCVB3	none	none	Cy3
**Figure S2**	mRNA	GFP (out of frame)	1.9	CleanCap and 100% N1Ψ	iCVB3	none	none	Cy3
**Figure S2, S3**	cirRNA	GFP (out of frame)	1.9	none	iCVB3	none	none	Cy5
**Figure S4**	cirRNA	GFP (out of frame)	1.9	none	iCVB3	none	none	Cy5
**Figure S1, S4**	cirRNA	GFP (out of frame)	1.9	none	iCVB3	none	none	AF488
**Figure S4**	linRNA	GFP (out of frame)	1.9	none	iCVB3	none	none	AF488
**Figure S4**	cirRNA	GFP (out of frame)	1.9	none	iCVB3	none	none	pHrodo
**Figure S4**	circRNA	NanoLuciferase	1.7	5% 2'-O-MethylIC	synIRES-RC25 (iHRVB3-based)	5′ PABP spacer	HBA1	none
**Figure S4**	mRNA	NanoLuciferase	1.7	CleanCap and 100% N1Ψ	none	5′ PABP spacer	HBA1	none
**Figure S6**	cirRNA	GFP (out of frame)	1.9	none	iCVB3	none	none	FITC
**Figure S6**	cirRNA	GFP (out of frame)	1.9	none	iCVB3	none	none	FITC
